# Effects of the Plant Growth-Promoting Bacterium *Burkholderia* *phytofirmans* PsJN throughout the Life Cycle of *Arabidopsis thaliana*

**DOI:** 10.1371/journal.pone.0069435

**Published:** 2013-07-15

**Authors:** María Josefina Poupin, Tania Timmermann, Andrea Vega, Ana Zuñiga, Bernardo González

**Affiliations:** 1 Laboratorio de Bioingeniería, Facultad de Ingeniería y Ciencias, Universidad Adolfo Ibáñez, Santiago, Chile; 2 Departamento de Ciencias Vegetales, Facultad de Agronomía e Ingeniería Forestal, Pontificia Universidad Católica de Chile, Santiago, Chile; Virginia Tech, United States of America

## Abstract

Plant growth-promoting rhizobacteria (PGPR) induce positive effects in plants, such as increased growth or reduced stress susceptibility. The mechanisms behind PGPR/plant interaction are poorly understood, as most studies have described short-term responses on plants and only a few studies have analyzed plant molecular responses under PGPR colonization. Here, we studied the effects of the PGPR bacterial model *Burkholderia* *phytofirmans* PsJN on the whole life cycle of *Arabidopsis thaliana* plants. We reported that at different plant developmental points, strain PsJN can be found in the rhizosphere and also colonizing their internal tissues. In early ontogeny, strain PsJN increased several growth parameters and accelerated growth rate of the plants. Also, an Arabidopsis transcriptome analysis revealed that 408 genes showed differential expression in PsJN-inoculated plants; some of these genes are involved in stress response and hormone pathways. Specifically, genes implicated in auxin and gibberellin pathways were induced. Quantitative transcriptional analyses of selected genes in different developmental stages revealed that the beginning of these changes could be evidenced early in development, especially among the down-regulated genes. The inoculation with heat-killed bacteria provoked a more severe transcriptional response in plants, but was not able to induce plant growth-promotion. Later in ontogeny, the growth rates of inoculated plants decreased with respect to the non-inoculated group and, interestingly, the inoculation accelerated the flowering time and the appearance of senescence signs in plants; these modifications correlate with the early up-regulation of flowering control genes. Then, we show that a single inoculation with a PGPR could affect the whole life cycle of a plant, accelerating its growth rate and shortening its vegetative period, both effects relevant for most crops. Thus, these findings provide novel and interesting aspects of these relevant biological interactions.

## Introduction

In the rhizosphere, plants and microorganisms are permanently interacting in a continuum ranging from deleterious (pathogens) to beneficial (symbionts) [1]. Extensive communication occurs between both parties during different stages of plant development, where signaling molecules from both actors play an important role. The final outcome of these relationships depends on the established molecular dialogue [2]. Among the beneficial interactions are those with plant growth-promoting rhizobacteria (PGPR) which colonize the rhizosphere or internal tissues of many plant species, inducing positive effects such as increased plant growth, reduced susceptibility to diseases (caused by fungi, bacteria, viruses and nematodes) and improved tolerance to abiotic stresses [1,3-6]. Different mechanisms of rhizobacterial growth promotion have been proposed. For instance, the ability to fix atmospheric nitrogen [7]; solubilization of inorganic nutrients that are rate-limiting for plant growth [8]; stimulation of nutrient delivery and uptake by plant roots; and the modulation of plant regulatory mechanisms through the production of hormones such as auxin, gibberellins and cytokinins [9-12], the reduction of plant ethylene levels [13-15] or the production of other compounds that influence plant development [16-18].

Although some studies report the effect of PGPR in the field [19,20], most of the studies using PGPR describe the effects of these bacteria in seedlings or in short-term periods [e.g. 21,22-24]. As PGPR could modulate plant regulatory mechanisms, an interesting question to be addressed is if these bacteria could also affect middle or late ontogenetic stages in plants. To our knowledge, only one study has reported long-term effects of a PGPR in Arabidopsis, assessing the effects of volatile organic compounds emitted by *B. subtilis* GB03 during the complete life cycle of plants [25].

Also, the mechanisms underlying PGPR-plant interactions, the genetic basis and signal transduction components that are involved in the growth promoting effects of PGPR in plants are scarcely understood. Unfortunately, only a few studies have reported transcriptional global changes in plants under PGPR colonization. Most of these studies have been focused on Induced Systemic Resistance (ISR) response to pathogens in *Arabidopsis thaliana* under PGPR colonization and report the effects of γ-proteobacteria such as *Pseudomonas* sp. [26-29]; gram positive bacteria such as *B. subtilis* [11], and even the photosynthetic α-proteobacteria *Bradyrhizobium* strain ORS278 [30]. Also, some recent efforts have been made to elucidate the transcriptional responses to PGPR of plants different from Arabidopsis [31]. These studies have revealed that transcriptional responses are highly dependent on the bacterial partner [1].

Some of the more diverse and environmentally adaptable plant-associated bacteria are β-proteobacteria belonging to the genus *Burkholderia* [32,33]. Bacteria of this genus can establish a wide range of relationships with plants. *Burkholderia* spp. can be free-living in the rhizosphere as well as epiphytic and endophytic, including obligate endosymbionts and phytopathogens [32,33]. *Burkholderia* *phytofirmans* PsJN is a PGPR able to produce positive effects in horticultural crops, such as tomato, potato and grape [34-39]. It has been reported that this bacterium stimulates growth of inoculated plants and induces physiological changes enhancing their adaptation to environmental stresses [34,40,41]. In addition, plants inoculated with strain PsJN present longer root systems, more secondary roots and root hairs; stronger stems and more lignin deposits on vascular bundles [42,43]. Also, inoculated plants present high amounts of phenolic compounds and chlorophyll content [34,42], high cytokinin levels [44] and a high phenylalanine ammonia lyase level [42]. Strain PsJN also enhances resistance to low levels of pathogens [45]. It has been proposed that the reduction of the plant ethylene hormone by the action of the strain PsJN enzyme 1-aminocyclopropane-1-16 carboxylate (ACC) deaminase could be involved in the induced plant growth-promotion [35,46]. Some recent efforts have been made to elucidate the molecular responses of plants under strain PsJN colonization, focusing on changes in specific stress response genes [40,47] or the methylation patterns of some plant’s genes [48]. However, global overviews of molecular changes that may explain the different effects of strain PsJN or other β-proteobacteria during plant development are not available.

Here, we report that a single inoculation of *A. thaliana* seeds with the strain PsJN, during germination, exerts phenotypic effects throughout the whole life cycle of the plants. We describe the changes in phenotype and transcriptional profiles of inoculated plants during early plant development and we compare the effects of live strain PsJN with those of heat-killed bacteria. We also describe the effects of this bacterium during later developmental stages where, interestingly, an acceleration of flowering time and senescence was observed in inoculated plants. This report provides novel and interesting information about long-term effects of a PGPR on plant development, contributing to the knowledge on these relevant biological interactions.

## Results

### Short-term effects of strain PsJN on *A. thaliana* plants

**Figure 1:**
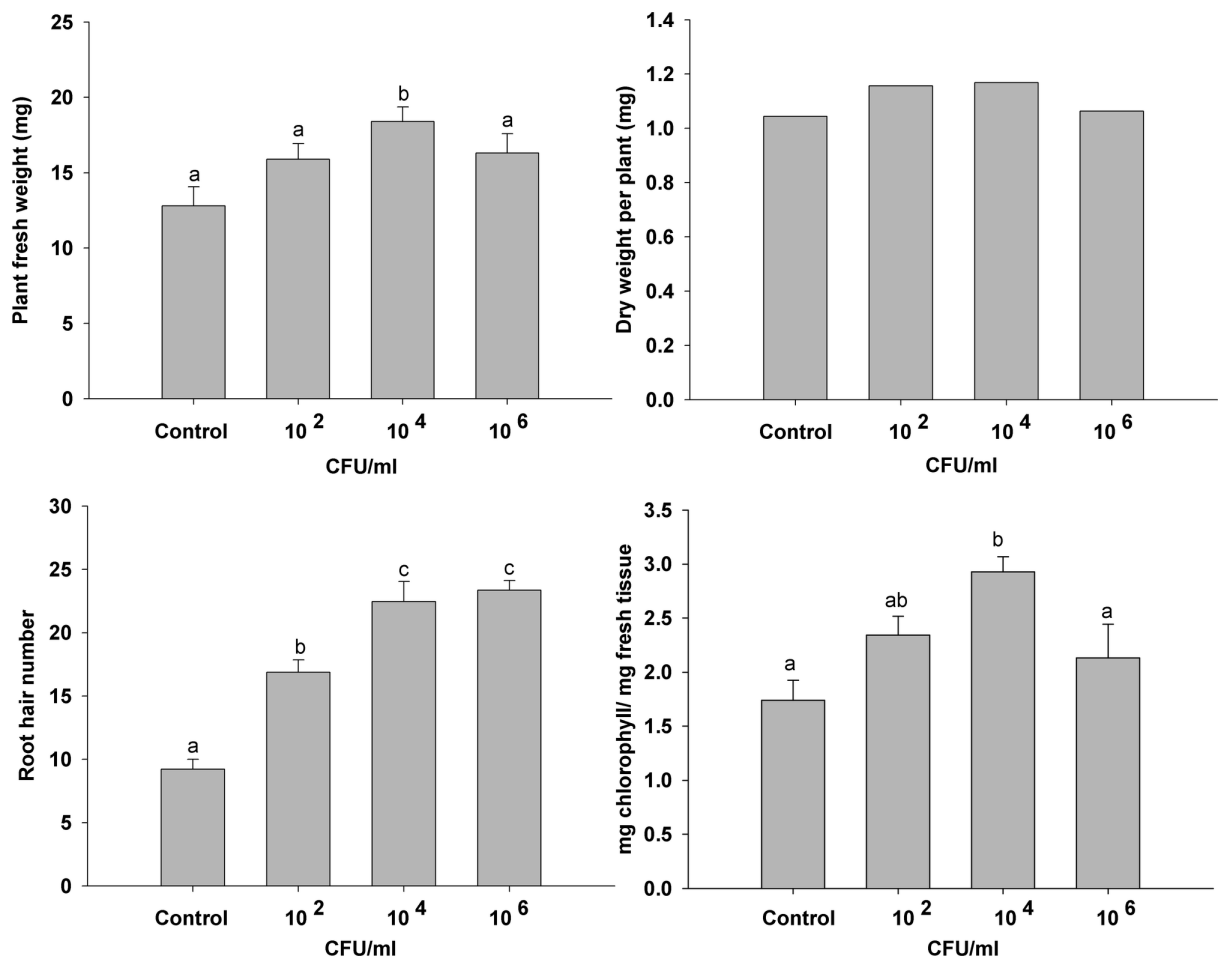
Short term effects of different inocula of *Burkholderia phytofirmans* PsJN in *Arabidopsis thaliana* plantlets. Different measurements in plants at 14 DAS treated with several bacterial inocula. A) Plant fresh weight, B) Dry weight, C) Root hairs and D) Chlorophyll content. Media and standard errors (SE) are the result of at least 40 plants per treatment and results are representative of three independent experiments. Same letters indicate non-significant differences among treatments (One way ANOVA, p<0.05).

**Figure 2:**
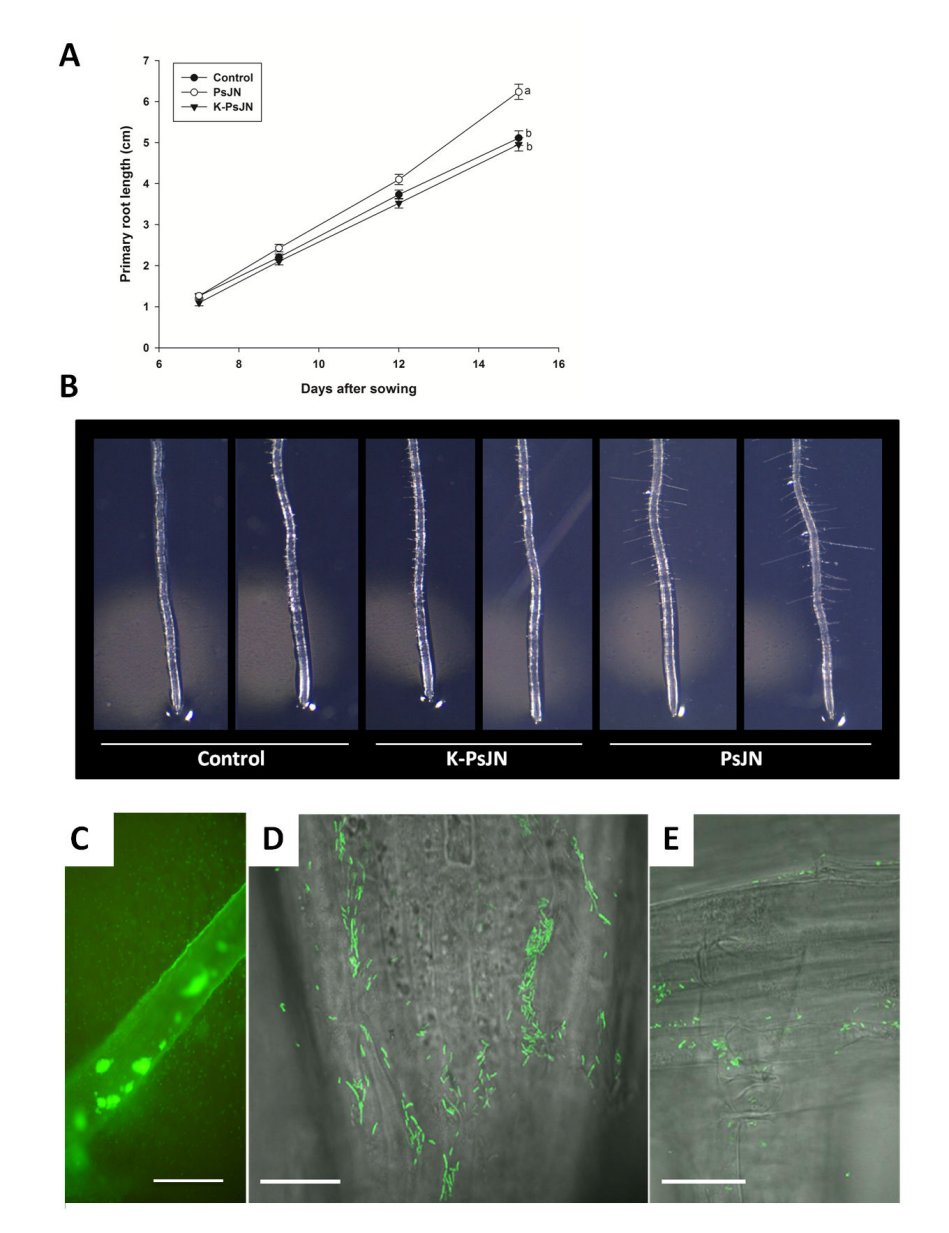
Effects of *Burkholderia* *phytofirmans* PsJN on *Arabidopsis* *thaliana* roots. A) *In vitro* growth of primary roots under different treatments. Media and SE are the result of at least 30 plants per treatment and results are representative of three independent experiments. Same letters indicate non-significant differences among treatments (One way ANOVA, p<0.05). B) Representative photographs of root hairs formed at the primary root tip region of 8 DAS Arabidopsis seedlings grown in indicated treatments. K-PsJN represents the killed bacteria treatment. C) Representative photograph of epifluorescence images of root colonization of *A. thaliana* plants by strain PsJN::GFP; bacterial cells are observed as colonies attached to the surface of the root (arrow), bar represents 200 μm. D) Confocal microscope image of a root colonization by strain PsJN::GFP at the root tip, bar represents 10 µm. E) Confocal microscope of a root colonized endophytically by PsJN :: GFP (roots were washed with ethanol and sterile water before being observed under microscope); cells are observed as colony attached to lateral root emergence and in an intercellular position, between the epidermal layer, bar represents 30 µm.

To look for differences in plant growth parameters, several PsJN strain dilutions (10^2^; 10^4^; 10^6^ colony forming units (CFU)/ml) were tested as inoculants of Col-0 *A. thaliana* seeds, as described in the Material and Methods section. At 14 days after sowing (DAS) several plant growth-parameters were determined (Figure 1). All parameters (plant fresh weight, dry weight, number of root hairs and chlorophyll content) were significantly higher than in the non-inoculated control, when 10^4^ CFU of strain PsJN/ml of medium were utilized. Also, a positive effect on hypocotyl length was observed in inoculated plants (data not shown). Therefore, the plant growth outcome at day 14 depended on the population of bacteria that was initially associated to plant. Essentially the same was observed at 21 DAS. Consequently, all the subsequent experiments were carried out using 10^4^ CFU of strain PsJN/ml medium. The effect of strain PsJN on root growth was further explored comparing root length at different times in plantlets growing vertically in non-inoculated and inoculated conditions. In addition, a treatment with heat-killed bacteria (K-PsJN) was incorporated to discriminate the effects of metabolically active bacteria on plants, from those of inactive bacteria. Roots were significantly longer at 14 DAS in inoculated plants (Figure 2A) (One-way ANOVA, p<0.001). At 14 DAS, no significant differences were detected on number of lateral roots among treatments. Root hairs were longer (250% longer in average compared with those of non-inoculated plants) and more abundant in strain PsJN-treated plants (Figure 2B). The non-inoculated controls and K-PsJN treatments produced equivalent results.

To determine the rhizospheric and endophytic colonization of plants by strain PsJN, a GFP marked strain was utilized. At 21 DAS rhizospheric CFU/mg of fresh weight (FW) were usually in the range of 10^9^-10^10^ (average 6.91x10^9^). Endophytic bacteria were detected on a range of 10^7^-10^8^ CFU/mg FW (average: 7.90x10^7^ CFU/mg FW). The same ranges were observed at 14 DAS (average of endophytic colonization: 1.28x10^7^ CFU/mg FW). Rhizospheric and endophytic colonization were confirmed by epifluorescence (Figure 2C) and confocal microscopy analyses (Figure 2D and 2E).

**Figure 3:**
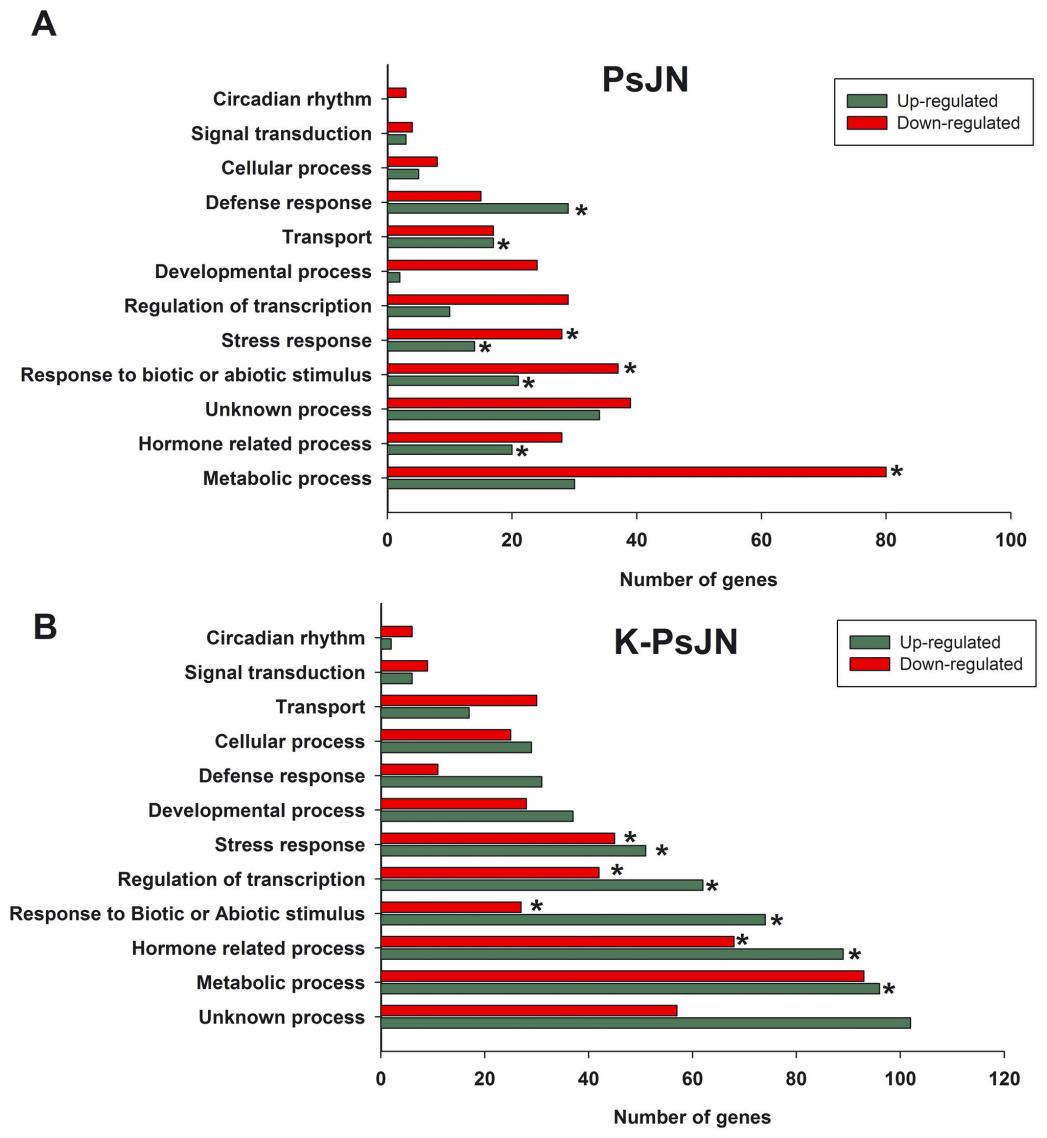
Gene ontology of biological processes affected in *Arabidopsis* *thaliana* plants treated with *Burkholderia* *phytofirmans* PsJN and K-PsJN. The figure shows the number of genes induced or repressed in plants treated by strain PsJN (A) or K-PsJN (B) in comparison to control conditions and its distribution within the different Gene Ontology biological functions. Only those genes with differential expression (p<0.05) were plotted, corresponding to 159 and 249 genes up-regulated and down-regulated, respectively in PsJN treatment. With the K-PsJN treatment 364 genes were up-regulated and 282 were down-regulated. Each gene could be assigned to more than one category. The VirtualPlant platform [50] was utilized in order to determine which GO terms were statistically overrepresented compared with the GO term represented into the Arabidopsis genome arrays (asterisks, p<0.01).

To investigate the molecular mechanisms underlying the phenotypical effects observed in plants treated with strain PsJN, transcriptional profiles were determined by microarray analysis (Affymetrix ATH1 Genome Array). Assuming that effects of strain PsJN on the Arabidopsis gene transcription profile take place before the major phenotypic changes are observed, the global gene expression patterns of complete plantlets with 4 rosette leaves (stage 1.04 [49], corresponding to 13 DAS), inoculated or not with bacteria, were compared. The presence of strain PsJN significantly up-regulated 159 genes and down-regulated 249 genes (RankProduct method; p<0.05). To get a global overview of these differentially expressed genes, the Gene Ontology categories (GO) that were present in the group of genes affected in the inoculated treatment were first analyzed (Figure 3A). The BioMaps tool, in the VirtualPlant platform [50], was utilized in order to determine which GO terms were statistically overrepresented compared with the GO terms represented into the Arabidopsis genome arrays. Several genes belonging to the biological processes “*Defense response*”, “*Stress response*”, “*Transport*”, “*Response to Biotic and Abiotic stimulus *” and “*Hormone related process*” were induced by the strain PsJN treatment (asterisk in Figure 3A). The transcriptionally down-regulated genes were statistically mostly distributed in the functional categories: “*Stress response*”, “*Response to Biotic and Abiotic stimulus*” and “*Metabolic Response*” (asterisks in Figure 3A). In the molecular functions categories, “*Unknown molecular functions*”, “*Binding activity*” and “*Kinase activity*” were overrepresented in the group of up-regulated genes. “*Transferase activity*”, “*Unknown functions*” and “*Binding activity*” were overrepresented in the down-regulated genes (Figure S1). Examples of genes with altered expression and with different biological functions are presented in Tables S1 and S2, and the complete list of genes affected by PsJN is in Table S3. Also, all the Affymetrix data obtained here have been deposited in NCBI’s Gene Expression Ommnibus [51] and are accessible through GEO Series accession number GSE47092. The gene expression changes of live PsJN strain treated plants were compared with those of K-PsJN inoculated plants (364 genes were up-regulated and 282 were down-regulated compared with the control); the GO categories of these genes are presented in Figure 3B. Interestingly, a minor percentage of these genes were also regulated by PsJN treatment (Figure S2). For instance, among the 159 genes up-regulated by PsJN, 105 were not regulated by the K-PsJN treatment (Figure S2). When the GO processes that are overrepresented were compared in PsJN and K-PsJN treatments by BioMaps tool, in the VirtualPlant platform, it could be observed that the transcriptional response is much more restricted with PsJN as more processes are affected in plants inoculated with K-PsJN (data not shown). Interestingly, the transcriptional changes produced by K-PsJN inoculation are not reflected in growth promotion or any other observable effects in plants. The complete list of genes affected by K-PsJN is available in Supporting Information (Table S4).

Several groups of Arabidopsis genes whose expression levels were altered by the presence of strain PsJN are especially interesting, e.g. genes related to development, transport, stress and hormone pathways. For example, genes involved in auxin pathways had altered expression: anthranilate synthase 1 (*ASA1*, At5G05730) which catalyzes the rate-limiting step of tryptophan biosynthesis; auxin indole-3-acetic acid (IAA) induced gene (*IAA1*, AT4G14560) belonging to the Aux/IAA transcription factor gene family; and the auxin responsive SAUR protein gene (*SAUR68*, At1G29510) were up-regulated in the inoculated plants. There were also down-regulated genes, which are related to the auxin pathway, as an auxin efflux carrier gene (At1G76520). A particularly interesting case of up-regulated gene is *AtGA3ox1* (*Gibberellin 3-beta-dioxygenase*, At1g15550), which catalyzes the final step in the synthesis of bioactive gibberellins. Among genes involved in stress or defense response we found genes involved in salicylic acid (SA) pathway, like *WRYK60* (At2G25000); *WRKY70* (At3G56400) and *WAK1* (At1G21250); and genes involved in jasmonic acid (JA) and ethylene pathways (e.g. *LOX2*, At3G45140 and *PDF1.2*, At5G44420), see Table S1, S2 and S3.

**Figure 4:**
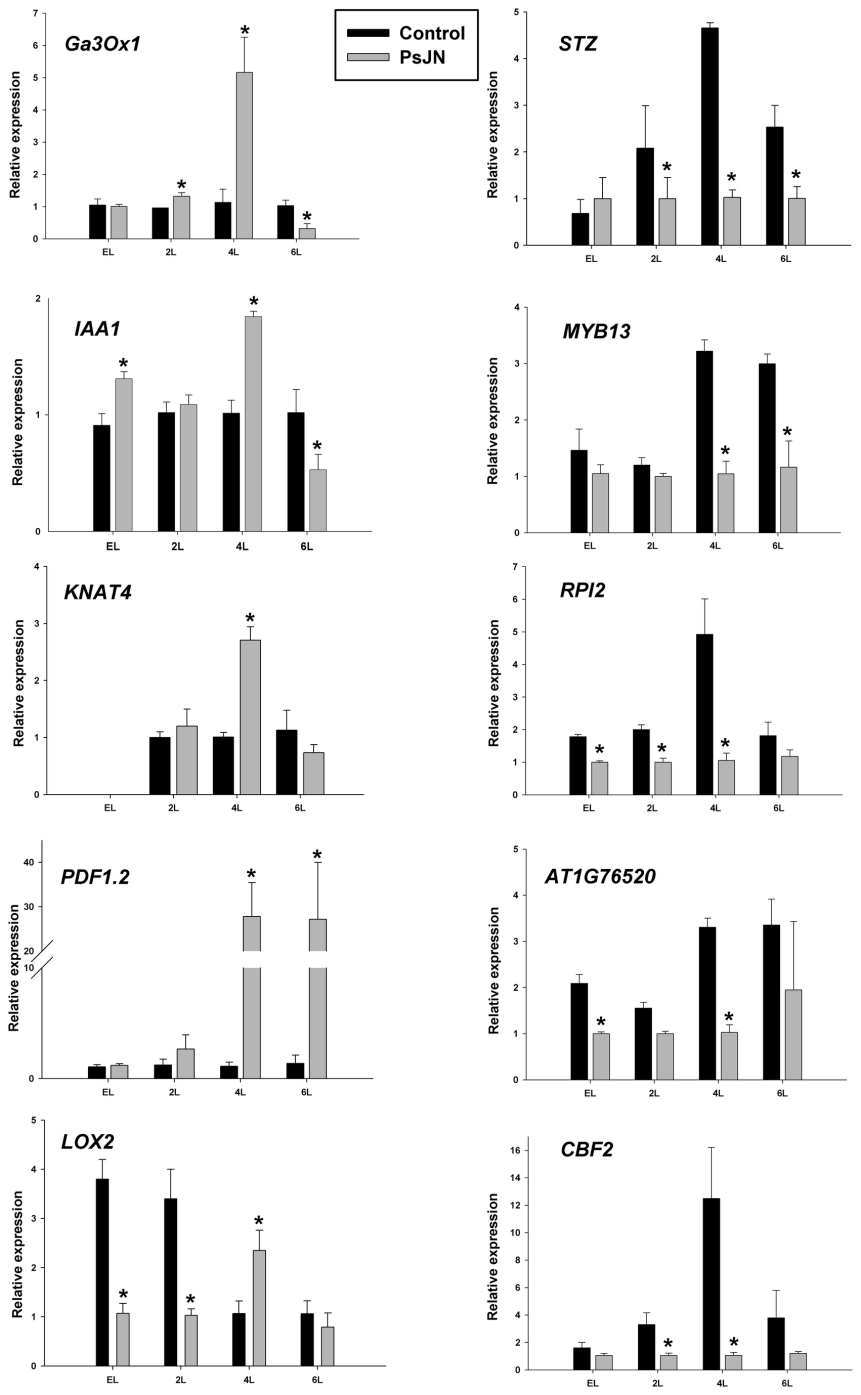
Quantitative real time PCR of selected up and down-regulated genes after inoculation of Arabidopsis with *Burkholderia* *phytofirmans* PsJN. Quantitative RT-PCR determinations of relative levels of gene expression in complete plants at 2 first leaves emerging (EL), 2 leaves expanded (2L), 4 rosette leaves (4L) or 6 rosette leaves (6L) stages. Graphics in the left side of the figure correspond to those genes that were up-regulated at 4L stage under PsJN treatment (the stage were affymetrix analyzes were performed) and in the right are those genes with down-regulation at 4L stage. Data are means ± SE. Asterisk indicates statistical significance (One way ANOVA, p<0.05).

Five up-regulated and five down-regulated genes, after inoculation with strain PsJN, corresponding to some of the biological functions overrepresented in the Affymetrix analysis, were selected for quantitative RT-PCR validation. These ten genes were confirmed as up or down-regulated after inoculation with live strain PsJN (Figure 4, at 4L stage). In some cases, the magnitude of the changes determined by quantitative RT-PCR was greater than those values from array analysis. To have a more comprehensive view of the transcriptional changes occurring in inoculated plants, expression of the same set of genes was evaluated in three additional time points (Figure 4). Thus, to evaluate whether the transcriptional changes observed in plants at 4 leaves stage under PsJN treatment began to appear earlier in the development, two early plant developmental stages were analyzed (emerging leaves, EL; and 2 leaves, 2L, corresponding to 7 and 11 DAS, respectively). Also, to assess whether those genes with transcriptional changes in the inoculated plants remained regulated over time, their transcriptional levels were analyzed in plants of 6 leaves (stage 1.06 [49],; corresponding to 19 DAS). Different patterns were found. For instance, 9 of the 10 tested genes presented severe transcriptional changes at 4L stage (the developmental stage were Affymetrix analyses were performed). In some of the up-regulated genes at 4L, the up-regulation started as soon as in EL stage (Figure 4, left panel). One of the up-regulated genes (*Lox2*) presented the opposite pattern of expression in earlier developmental stages (being down-regulated in PsJN treatment in EL and 2L stage; Figure 4). Notably, the five down-regulated tested genes presented the same pattern, being down-regulated since the first analyzed stage (Figure 4, right panel).

Regarding the last developmental stage analyzed, it was found that among the up-regulated genes most of them turned to be down-regulated at 6L (Figure 4, left panel). As example, *Ga3Ox1* was up-regulated by strain PsJN treatment at 4 leaves stage, but down-regulated at the 6 leaves stage (Figure 4, left panel). Only the defense-response related gene *PDF1.2* remained up-regulated at the 6 leaves stage (Figure 4, left panel). In contrast, among the genes down-regulated at 4 leaves stage, most of them were still down-regulated at this later stage (Figure 4, right panel).

### Long-term effects of strain PsJN on *A. thaliana* plants

**Figure 5:**
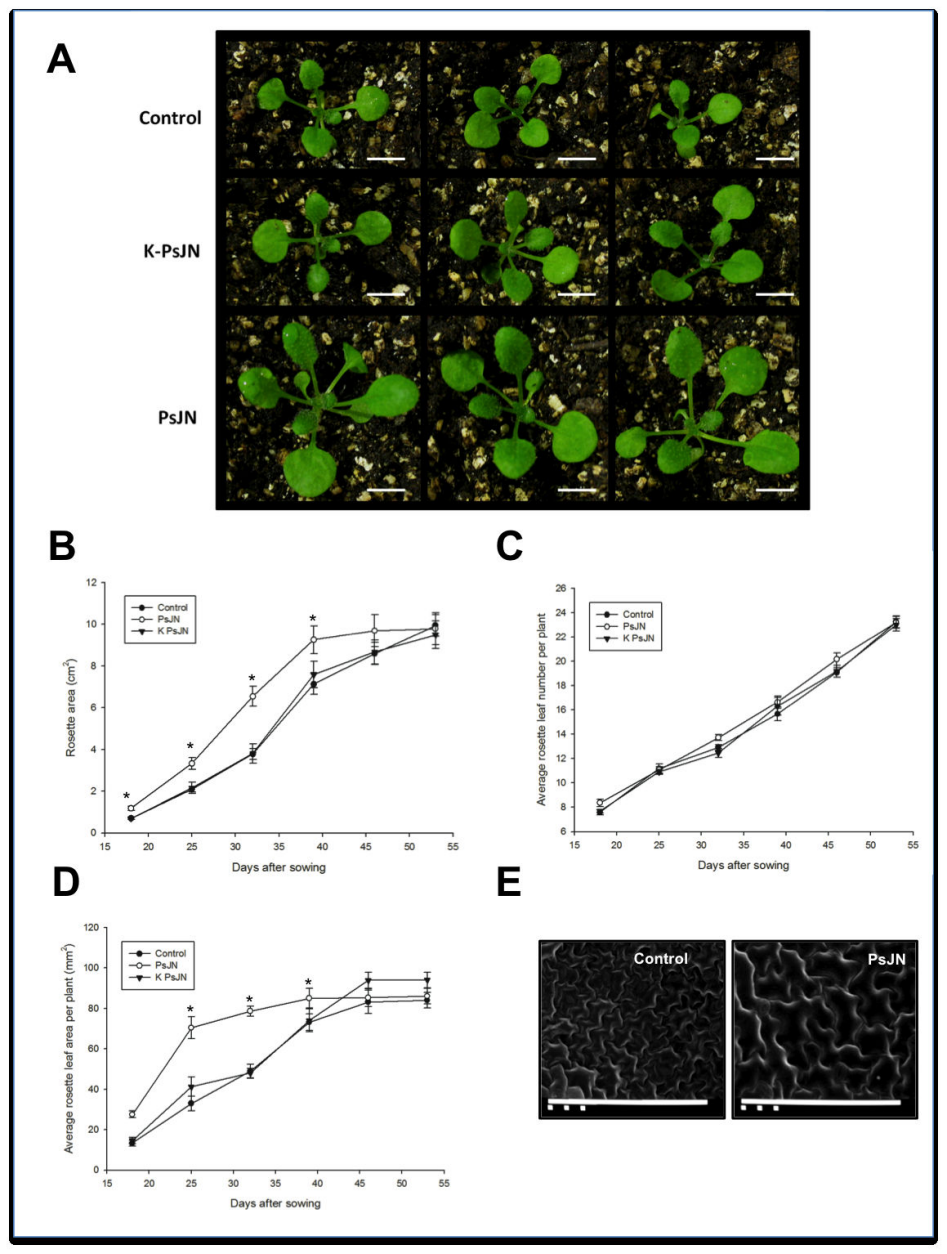
Effects of *Burkholderia phytofirmans* PsJN on aerial zone during long-term growth of *Arabidopsis thaliana* plants. A) Representative photographs of *A. thaliana* rosettes of plants exposed to the different treatments (K-PsJN represents the killed bacteria treatment) at 18 DAS, bars correspond to 0.5 cm. B–D) Graphic representation of rosette media areas (B) average rosette leaf number per plant (C) and average rosette leaf areas (D) of plants subjected to the different treatments. Media and SE were calculated with at least 12 plants per treatment, and results are representative of three independent experiments. Asterisks indicate significant differences among PsJN treatment and the two other treatments in each time (One or two-way ANOVA, p<0.05). E) Representative photographs of the adaxial surface of third rosette leaves from 40 DAS plants. Bars represent 100 µm.

Most of the studies on interactions of PGPR-plants have addressed short-term effects on *in vitro* plants. To evaluate the effect of strain PsJN in middle and late plant ontogenetic stages, inoculated plantlets were transferred to a 2:1 mix of peat/vermiculite substrate at 14 DAS. The aerial zone of these plants was digitally photographed and the images were processed to compare the different treatments (Figure 5A). Figure 5B shows that rosette areas in inoculated plants were larger over most of the recorded period. However, at the end of the measurements, at 53 DAS, rosettes of all treatments reached very similar areas (Figure 5B). The growth rates of rosettes over the first 14 days on substrate were 0.21±0.02, 0.37±0.03 and 0.21±0.03 cm^2^/day for non-inoculated, inoculated and K-PsJN treatments, respectively. Thus, strain PsJN-treated plants had significantly higher growth rates compared to the other treatments (One way ANOVA, p=0.01). Nevertheless, when the same rates were compared over the following 21 days, non-significant differences were observed: 0.29±0.02; 0.19±0.03 and 0.27±0.03 cm^2^/day for control, strain PsJN and K-PsJN treatments, respectively. An analysis of covariance separate slopes model corroborated that strain PsJN had different effects on rosette growth rate, depending on the stage of the experiment (one-way ANCOVA, p<0.0001) (Figure S3). Then, strain PsJN-treated plants had higher growth rates in the first stages of growth, whereas at the end of the experiment rates tended to be similar among all treatments, to a point where the growth rates of the three treatments reached almost the same value (Figure S3). The average number of leaves per plant did not show significant differences across all treatments (Figure 5C; Two-way ANOVA, p =0.87405), although the averages of rosette leaf areas where significantly larger in the PsJN-treated plants, over most of the recorded period (Figure 5D; Two-way ANOVA, p<0.0001). Thus, to test if the accelerated growth and the larger sizes of leaves provoked by strain PsJN could be explained by cell expansion, a scanning electron microscopic examination was performed to the adaxial surface of third rosette leaves from 40 DAS plants, in a point where rosette areas and leaf sizes were still larger in inoculated plants than in the control group. It was observed that the average epidermal cell size was significantly higher (One way ANOVA, p<0.005) in plants treated with bacteria (Figure 5E): 704±69 µm and 896±52 µm in control and strain PsJN treatments, respectively. This indicates that at least at this time, strain PsJN probably stimulates cell growing and elongation, although an additional effect regarding cell division cannot be ruled out. Additionally, at 40 DAS, rhizospheric CFU/mg FW were at a level of 10^4^ (average: 3.43x10^4^ UFC/mg FW). At this time, PsJN strain was found in the internal tissues of the aerial zone of the plants at a level of 10^3^ (3.07x10^3^ UFC/mg FW average).

**Figure 6:**
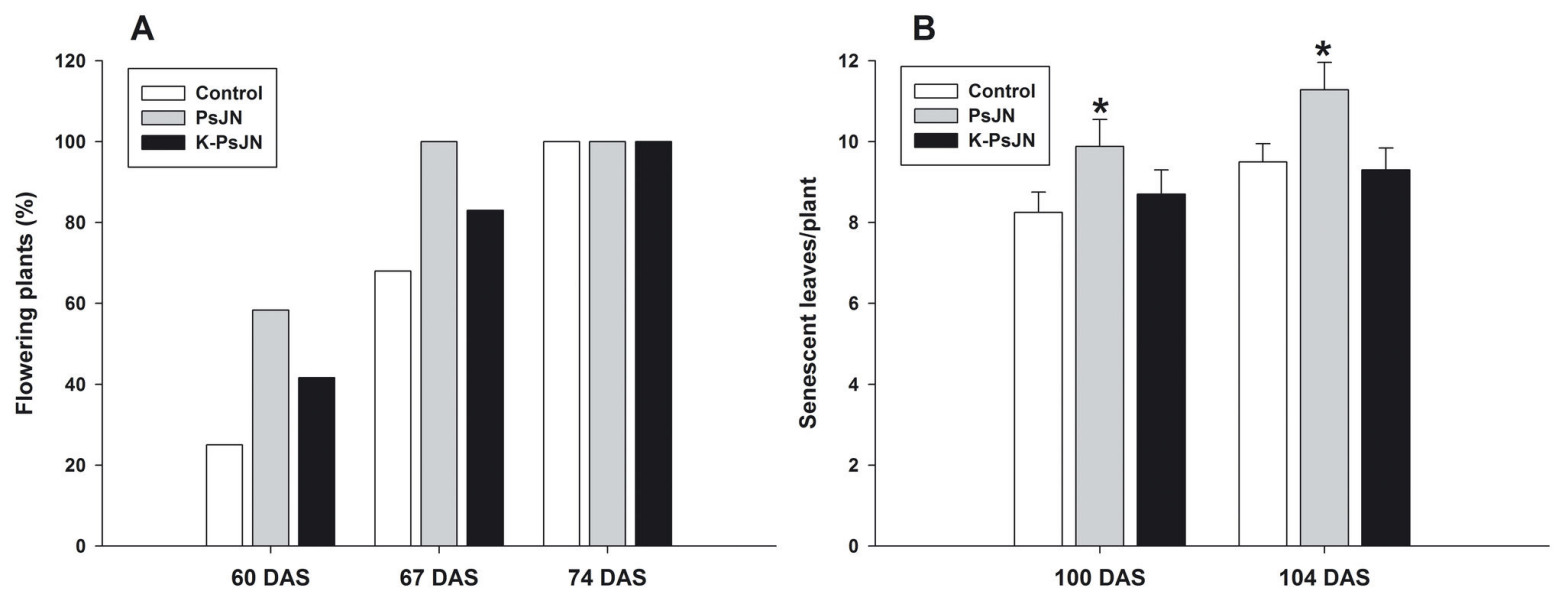
Effects of *Burkholderia* *phytofirmans* PsJN on flowering and senescence of *Arabidopsis thaliana* plants. A) Percentage of plants presenting floral primordia in the different treatments. B) Number of senescent leaves in plants of the different treatments at different days after sowing. Asterisks represent significant differences (One way ANOVA, p<0.05). Media and SE represent measurements of at least 12 plants per treatments and results are representative of three independent experiments.

**Figure 7:**
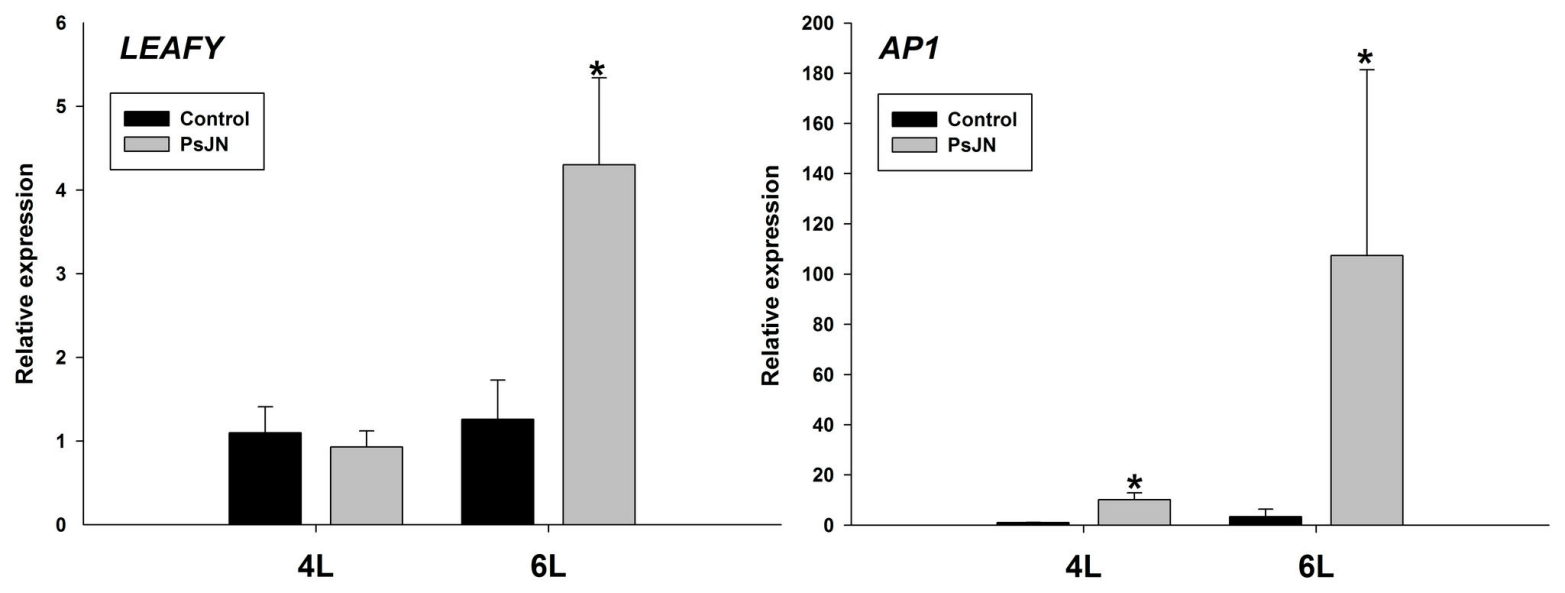
Expression of flowering key regulator genes after inoculation of Arabidopsis with *Burkholderia* *phytofirmans* PsJN. Quantitative RT-PCR determinations of relative levels of expression of *LEAFY* and *AP1* (*APETALA1*) genes in complete plants at 6 rosette leaves stage. Data are means ± SE. Asterisk indicates statistical significance (One way ANOVA, p<0.05).

Notably, flowering time was also affected in strain PsJN-treated plants, since at 60 DAS 58.3% of inoculated plants presented floral primordia, while the control and K-PsJN-treated plants presented 25 and 41.6%, respectively. A similar pattern was observed at 67 DAS and plants of all treatments presented at least one flower at 74 DAS (Figure 6A). The same pattern was observed under different photoperiod schemes (data not shown). Also, senescence was observed earlier in strain PsJN-treated plants. When the number of senescent leaves per plant was recorded at 100 and 104 DAS, live strain PsJN-treated plants presented significantly higher (One way ANOVA, p<0.005) values than the other two treatments (Figure 6B). The averaged seed number per silique did not show significant differences among all treatments (data not shown). To test if the effects that were observed later in the ontogeny of inoculated plants, like the early flowering, correlated with changes on gene expression in early ontogeny, we evaluated the expression of the key flowering regulators genes *LEAFY* (*LFY*, At5G61859) [52-54] and *APETALA1* (*AP1*, At1G69120) [55]. Interestingly, we found that in inoculated plants AP1 was up-regulated at 4L stage and that both genes were significantly up-regulated in inoculated plants at 6L stage (Figure 7).

## Discussion

We found that *B* *. phytofirmans* PsJN induced different positive effects on *A. thaliana* plants. A single event of inoculation during the germination phase had effects on growth parameters during the early and late stages of plant development. The first observable effects of bacteria in plants were changes in root length and a higher number and length of root hairs. Also, higher plant weight and chlorophyll content was found in these inoculated plants. Different initial bacterial concentrations were tested, inducing different levels of plant responses. Diverse effects depending on the initial concentration of PGPR have also been reported by Belimov et al. [56]. They showed that a single PGPR strain ( *Pseudomonas* *brassicacearum* Am3) produces growth-promoting, neutral, or phytopathogenic effects on a single plant cultivar (tomato), according to the inoculation dose and environmental conditions [56].

We reported that strain PsJN developed abundant both rhizospheric and endophytic populations in Arabidopsis plants, even in the aerial tissues, which has been also described for this bacterium in grapevine [35,57]. A stimulating question that arises from these observations (which is experimentally challenging), is whether the genetic or phenotypic changes found at later stages are, all or in part, due to the early contact of the plant with this bacterium, are provoked by the bacterial cells that currently thriving on plant compartments, or are explained by a mixture of both situations.

The genetic basis and signal transduction components that mediate the growth promoting effects of PGPR in plants are scarcely understood, as only a few studies have reported global transcriptional changes under PGPR colonization [11,26,28-31]. As far as we know, this is the first report of global changes in gene transcription of plants, induced by a β*-*proteobacterium.

Plant hormones play a crucial role in plant development. In this study, several genes involved in hormone pathways were detected with an altered expression level. Auxin is implicated in a variety of plant developmental processes, including lateral root development and elongation of the hypocotyls [58-60]. It has been described that fine tuning of auxin and cytokinin concentrations must be established among the root system and the aerial part of plants [61]. Therefore, it is not surprising that genes involved in homeostasis of both hormones are up and down-regulated in inoculated plants (Table S1, S2 and S3). Interestingly, our research group has recently found that indole-3-acetic acid (auxin, IAA) mineralization produced by strain PsJN plays a key role in plant growth-promoting traits and it is necessary for efficient Arabidopsis rhizosphere colonization. Also, using a transgenic *A. thaliana* line with suppressed auxin signaling (miR393), we have found that auxin signaling in plants is necessary for the growth promotion effects produced by the strain PsJN [62], which is well correlated with the Affymetrix results reported here.

Bioactive gibberellins (GAs) are a major class of phytohormones that regulate plant growth and development, from seed germination and vegetative growth to fruit and seed set [63,64]. Among the up-regulated genes in strain PsJN-treated plants, one of particular interest is *GA3ox1* (Gibberellin 3-beta-dioxygenase, At1g15550), which is involved in the synthesis of bioactive gibberellins. *GA3ox1* is responsible for the synthesis of bioactive GAs during vegetative growth [65] and it is involved in the determination of rosette areas and flowering time [66]. We report here that *GA3ox1* is up-regulated in inoculated plants and, in agreement, these plants present bigger rosette areas and early flowering times. Experiments using *ga3ox1* mutants inoculated with strain PsJN are required to further investigate the role of this gene on the responses of Arabidopsis to strain PsJN. Here we use complete plantlets for RNA extraction; future experiments that consider the analysis of spatial expression patterns of this gene (discriminating among the different tissues of plants), and the other interesting genes found in this work whose expression depend of live strain-PsJN inoculation, are also required to properly explain the phenotypes observed in plants. For example, such studies are required in the case of genes presumably involved in strain PsJN-induced plant protection to environmental or biotic stresses [40,41,45]: overexpressed genes involved in the salicylic acid (SA) pathway, like *WRKY60* (At2G25000), *WRKY70* (At3G56400) and *WAK1* (At1G21250); and *LOX2* (At3G45140) and *PDF1.2* (At5G44420) genes involved in jasmonic acid (JA) and ethylene pathway.

Additionally, we described that when PsJN is inactivated by heat, more drastic transcriptional changes are observed. However, these changes in transcription are not able to induce observable phenotype in plants. The transcriptional profiles of plants inoculated with both treatments were quite different and a particular group of genes that is regulated only by the live bacteria was found (see also Figure S2). This suggests that metabolically active cells are required to induce particular transcriptional changes in plants that are correlated with growth promotion. This is in agreement with studies that have reported a reduction of plant growth-promotion effects when PsJN strains, mutated in specific genetic pathways, are used to colonize [62,67,68].

Furthermore, we selected a group of genes with altered expression under PsJN inoculation and measured their relative expressions at different plant developmental stages. We found that in most cases the more drastic changes were observed at 4L stage, that in some cases changes in gene expression induced by strain PsJN were observed early in plant development (first emerging leaves stage, EL), and that, especially among the down-regulated genes, the down-regulation was observed over all the measured points.

Strain PsJN accelerates growth rate during the first half of plant development, then the growth rates level off and size converges with non-inoculated plants. We demonstrated that the larger rosette areas of inoculated plants in the first half of their life cycles are related with larger leaf areas rather than a major number of leaves. Therefore, at least under our experimental conditions, strain PsJN acts as a PGPR accelerating the growth rate rather than producing bigger plants.

The last could be related with the accelerated flowering and senescence time observed in inoculated plants. The controversial rate of living theory, a theory of aging mainly developed in animals, proposes that longevity is negatively correlated with metabolic rate [69,70]. Also in animals, studies in several taxa indicate that fast growth *per se* may have both negative and positive effects. There appears to be a link between accelerated growth and lifespan: rapid growth early in life is associated with impaired later performance and reduced longevity [71]. The faster growth observed in early stages of inoculated plants could be explained by different possible effects of bacteria, which may result in a better availability and acquisition of nutrients and/or a direct effect on plant metabolism. Ait Barka et al. [34] reported enhanced photosynthetic activity in strain PsJN inoculated grapevine plants exposed to two different temperatures, demonstrating that inoculation with strain PsJN may enhance this trait in plants. Measurements of metabolic rates in strain PsJN inoculated plants are required to elucidate if this trait may explain, at least in part, the early appearance of aging signs in inoculated plants.

To our knowledge, only one study has been reported long-term effects of a PGPR in Arabidopsis [25], i.e. that a long-term exposure to volatile compounds continually produced by the PGPR *B. subtilis* GB03 enhanced growth of this plant. In contrast with the shortened vegetative phase reported here, such a different PGPR and experimental scheme resulted in a delayed flowering in Arabidopsis [25].

Understanding the mechanisms behind PGPR-plant interactions is important to improve strategies for the use of these beneficial bacteria in agriculture. Here, we showed that a PGPR might affect the whole life cycle of a plant, accelerating its growth rate and shortening its vegetative period, both effects relevant for most crops. Also, we described that PsJN affects the expression of several genes early in Arabidopsis development, where the regulation of genes involved in auxin and gibberellins pathways may explain, at least in part the observed phenotypical changes upon inoculation. Further analyses will be useful to confirm the importance of these candidate genes in the growth promotion exerted by strain PsJN. Apparently, the strain PsJN effects on gene expression are less severe in the long term after inoculation, suggesting that early changes on gene expression could be involved in the phenotypes that are observed later in plant ontogeny. For instance, the expression of key regulator genes is up-regulated early in the development of inoculated plants and could explain the accelerated flowering time observed in the treated plants. Overall, these findings contribute to a better understanding of plants and beneficial bacteria interactions and provide novel information of the long-term effects of a PGPR on plant development, opening new avenues to study these relevant biological associations.

## Materials and Methods

### Plant growth conditions and treatments

*B* *. phytofirmans* PsJN, kindly provided by A. Sessitsch (AIT, Austria), was routinely grown in minimal saline medium [72], containing 10 mM fructose, in an orbital shaker (150 rpm) at 30°C. Cell suspensions from each inoculum were then collected and adjusted to approximately 10^8^ colony forming units per millilitre (CFU/ml), as determined by plate counting. Col-0 *A. thaliana* seeds were obtained from the ABRC. Seeds were surface sterilized with 50% sodium hypochlorite (100% commercial laundry bleach) containing 0.1% Tween 20, rinsed three times with sterile water, and kept at 4°C for 4 days to synchronize germination. Then, seeds were sown on square Petri dishes with half strength Murashige and Skoog medium (MS) 0.8% agar [73], inoculated or not with different dilutions of strain PsJN (10^2^; 10^4^ and 10^6^ CFU/ml). To assess the effect of inactivated bacteria, an inoculum was heated at 95°C for 20 min and then was used at a dilution of 10^4^ CFU/ml. Mortality was corroborated by plate counting. Plates were placed vertically in a growth chamber at 22°C with a photoperiod of 12 h of light and 12 h of dark. At day 14 after sowing (14 DAS) different growth parameters were determined in plants. For the transplanting experiment, seeds were sown and inoculated as described before, and after 14 days plantlets were transferred to individual pots with a 2:1 mix of peat/vermiculite and maintained at the same environmental conditions. Plants were watered with sterile water twice a week.

### Microscopy and image analysis

For scanning electronic microscopy analysis, leaves of the third line of the rosette [74] were fixed in cacodylate-buffer 1% (pH 7.2) and 3% glutaraldehyde for 24 h at room temperature according to Lartey et al. [75]. Subsequently, samples were dehydrated in a series of ethanol washes followed by 100% acetone. Samples were critical point-dried, sputter-coated with gold and observed under a Jeol JSM-25-SII scanning electron microscope. All images were analyzed using the ImageJ software.

### Determination of rhizospheric and endophytic bacterial colonization

For rhizoplane colonization tests, plants at 14 or 21 DAS were removed from the inoculated agar and were washed in phosphate buffer solution, with vortex agitation. The extracted liquid material was serially diluted with Dorn mineral salts medium before plating on Dorn medium plates supplemented with benzoate as the sole carbon and energy source. The colony forming units per milligram of fresh weight (CFU/mg FW) were determined after 48 h of incubation at 30°C. For endophytic colonization tests, plantlets inoculated with GFP-labeled PsJN strains were removed from the agar plates, and surface sterilized with 70% ethanol for 1 min, followed by 1% commercial chlorine bleach and a 0.01% Tween 20 solution for 1 min, and then washed three times in sterile distilled water (adapted from Compant et al. [35]). Plating the distilled water from a final wash on R2A medium routinely controlled sterility on these plants. Then, the sterilized plant material was macerated in sterile mortars and the disrupted tissue was resuspended in 1 ml of sterile 50 mM phosphate buffer to obtain an aqueous extract. CFU/mg FW were determined by serial dilutions of these extracts in R2A agar plates after 48 h of incubation at 30°C and examined under UV light using an Optical Epi-fluorescence Nikon Eclipse 50i microscope (Nikon, Japan). Confocal microscope images were obtained using Olympus FluoView 1000 confocal laser scanning (Olympus, Japan) equipped with high performance sputtered filters to examine fresh roots from inoculated plantlets with PsJN: GFP. For the analysis at 40 DAS, plants growing in pots (Phytatray 1552, Sigma-Aldrich, USA) were removed from the inoculated agar and the rhizospheric population was measured as described above. To test the presence of PsJN on aerial tissues, plants inoculated with PsJN strain were removed from the agar pots, the roots were removed and the aerial zones were sterilized to measure the colony forming units, as mentioned before.

### Plant growth measurements and statistical analysis

Fresh and dry weight of plants was determined with a Shimadzu analytical balance (Shimadzu Corporation, Japan). The chlorophyll contents were determined following a published procedure [76]. Chlorophyll was extracted from leaves of *A. thaliana* in N, N-9-dimethylformamide for 24 h at 4°C in dark, and chlorophyll a and chlorophyll b concentrations were measured simultaneously by spectrophotometry [76]. Growth of primary roots was registered using a rule. For dry weight measurements, plants for each treatment were grouped and then dried at 65°C for 24 h. The number and length of root hairs was analyzed in the same segment from the root tip with a stereo microscope (Leica S6D, Germany), considering the first portion of the root that presented root hairs over the meristematic region.

The plants in soil were photographed every seven days, starting four days after transplantation; rosette and leaf areas were calculated using the ImageJ software. Flowering plants were registered as those presenting a visible floral primordium. Senescent leaves were considered as those with at least 1/3 of their area with senescence signs.

To test for significant differences in response variables, one-way or two-way ANOVA were performed, using Kolmogorov–Smirnov and Cochran tests for normality, and Hartley and Bartlett tests for homogeneity of variances. Statistical analyses were carried out using the General Linear Models option in the statistical software package STATISTICA (version 6.0; StatSoft Inc., Tulsa, OK). When differences in the means were significant, a Tukey’s HSD test was performed [77]. A Bonferroni correction was applied to adjust significance levels for multiple comparisons. Cell and rosette area data were not normally distributed (p<0.05) and were Log_10_ transformed [77]. An ANCOVA separate slopes model test was used to analyze the effect of treatments (strain PsJN and K-PsJN) and time regarding the growth rates of rosettes. Tukey’s HSD multiple comparison test with Bonferroni correction was applied to determine which treatments were significantly different from others.

### RNA extraction, cDNA synthesis and qRT-PCR analyses

For RNA extraction, plants with the first rosette leaves visible emerging (EL; corresponding to 7 DAS under our experimental conditions), with 2 leaves expanded (2L, corresponding to 11 DAS), with 4 visible leaves (4L; LP.04 stage [49], corresponding to 13 DAS) and with 6 visible leaves (6L; LP.06 stage [49], corresponding to 19 DAS) were used; about 10 plantlets for each treatment were collected in liquid nitrogen and ground with a pestle in an Eppendorf tube. Then, RNA was obtained using the Trizol® (Invitrogen™, USA) method following the manufacturer’s instructions. For cDNA synthesis, 1 μg of total RNA treated with DNAse I (RQ1, Promega, USA) was reverse transcribed with random hexamer primers using the Improm II reverse transcriptase (Promega, USA), according to the manufacturer’s instructions. Real time (RT)-PCR was performed using the Brilliant® SYBR® Green QPCR Master Reagent Kit (Agilent Technologies, USA) and the Eco Real-Time PCR detection system (Illumina®, USA) as described by Poupin et al. [78]. The PCR mixture (15 μl) contained 2.0 μl of template cDNA (diluted 1:10) and 140 nM of each primer. Amplification was performed under the following conditions: 95°C for 10 min, followed by 40 cycles of 94°C, 30 s; 60-64°C, 30 s; and 72°C, 30 s, followed by a melting cycle from 55°C to 95°C. Relative gene expression calculations were conducted as described in the software manufacturer’s instructions: an accurate ratio between the expression of the gene of interest (GOI) and the housekeeping (HK) gene was calculated according to equation: 2-^(∆CtGOI-HK)^ [79]. Then, gene expression levels were normalized to the average value of the treatment with less expression. Expression of three housekeeping genes was analyzed for treatments *AtSAND* (AT2G28390), *PP2A* (AT1G13320) and *TIP41-like* (AT4G34270), using described PCR primer pairs [80,81]. In all cases, expression of HK genes was highly stable and similar results were obtained using them as normalization genes. Data presented here represent the normalization using *AtSAND* amplification. Primers designed in this study were designed using Primer Express v.2.0 (Applied Biosystems, USA) and confirmed with Primer-BLAST (NCBI). Sequences of all primers and their references (if applicable) are listed in Table S5. In all cases the reaction specificities were tested with melt gradient dissociation curves and electrophoresis gels (agarose 2%) of each PCR product. All experiments were performed with three to five biological and two technical replicates.

### Microarray hybridization

Three biological replicates, consisting of ten plantlets of 13 DAS each, for control and strain PsJN treatments, were used for global gene expression analysis using the GeneChip® Arabidopsis ATH1 Genome Array (Affymetrix®, USA). RNA samples were quantified and analyzed in terms of their quality by NanoDrop™ (Thermo Scientific, USA) spectrophotometer, according to the manufacturer’s instructions. RNA samples were further processed with the GeneChip 3’ IVT Express Kit aRNA amplification (Affymetrix®, USA), according to manufacturer’s directions. Single-stranded cDNA synthesis was performed with 0.5 μg of RNA of each sample, using oligo-dT-T7 Promoter Primer and the Superscript II reverse transcriptase (Invitrogen™, USA). Subsequently, double-stranded cDNA was synthesized and used as a template to generate biotinylated-targeted aRNA (cRNA), following the manufacturer’s specifications. Fifteen micrograms of the biotinylated aRNA was fragmented between 35 and 200 bases in length. The fragmented aRNA (10 μg) was hybridized on a GeneChip® Arabidopsis ATH1 Genome Array using standard procedures (45 °C for 16 h). The arrays were washed and stained in a Fluidics Station 450 (Affymetrix®, USA).

### Affymetrix data processing and analysis

The chip is composed of approximately 22.500 *A. thaliana* probe sets and was designed in collaboration with The Institute for Genome Research (TIGR). Data from the TIGR database (ATH1-121501) are available in the NetAffxTM Analysis Center. Array scanning was carried out with the GeneChip® scanner 300 and image analysis was performed using the GeneChip® Operating Software. GeneChip® arrays data were first assessed using a set of standard quality control steps described in the Affymetrix manual “GeneChip® Expression Analysis: Data Analysis Fundamentals”. Calls of all three spike-in controls BioC, BioD, and Cre were present, and their intensity values increased from BioC to Cre, as expected. Average background values ranged from 25 to 26. Digestion curves displaying trends in RNA degradation between the 5’ and 3’ end in each probe set were also inspected and all behaved in a similar manner.

Array data was processed and normalized by RMA (Robust Multi-Array Average) [82] using the R package known as “affy” [83]. Pearson rank coefficients were computed on the RMA expression values (log_2_-transformed) for each set of biological replicates. Pearson coefficients ranged between 0.98 and 0.99. The data discussed in this publication have been deposited in NCBI’s Gene Expression Omnibus [51] and are accessible through GEO Series accession number GSE47092. Differentially expressed genes were identified using RankProduct method [84]. Genes with a p<0.05 were identified as differentially expressed genes between treatments and were selected for further analysis. For functional analysis, we used the VirtualPlant platform [50]. In order to determine which Gene Ontology terms were statistically overrepresented, the BioMaps tool in this platform was used with a p-value cut-off of 0.01. For those Affymetrix IDs that represents more than one loci, all loci were considered for further analyses.

## Supporting Information

Figure S1 Molecular functions affected by strain PsJN and K-PsJN treatments. Molecular functions of the up-regulated genes or down-regulated genes under the different.
(TIF)

Figure S2 Changes in gene expression in PsJN and K-PsJN-treated plants. Venn diagrams of up-regulated and down-regulated genes in complete plants of 4 rosette leaves stages under PsJN or K-PsJN treatments. The intersections show the number of genes that are.
(TIF)

Figure S3 Lineal regression between rosette area and days after sowing, under the different treatments. Data were Log_10_ transformed, and each circle or triangle represents data from one plant.
(TIF)

Table S1 Examples of up-regulated genes under PsJN treatment classified in different functional categories*. (DOCX)

Table S2 Examples of down-regulated genes under PsJN treatment classified in different functional categories*. (DOCX)

Table S3 Complete list of genes that are regulated by PsJN treatment. (XLSX)

Table S4 Complete list of genes that are regulated by K-PsJN treatment. (XLSX)

Table S5 List of real time RT-PCR primers. Melting temperature and references, if applicable, are indicated.
(DOCX)
