# Supplementary figures and images for: Effects of the Plant Growth-Promoting Bacterium Burkholderia phytofirmans PsJN throughout the Life Cycle of Arabidopsis thaliana
Source: PLoS One. 2013 Jul 15;8(7):e69435. doi: 10.1371/journal.pone.0069435 (PMC3711820; doi:10.1371/journal.pone.0069435)

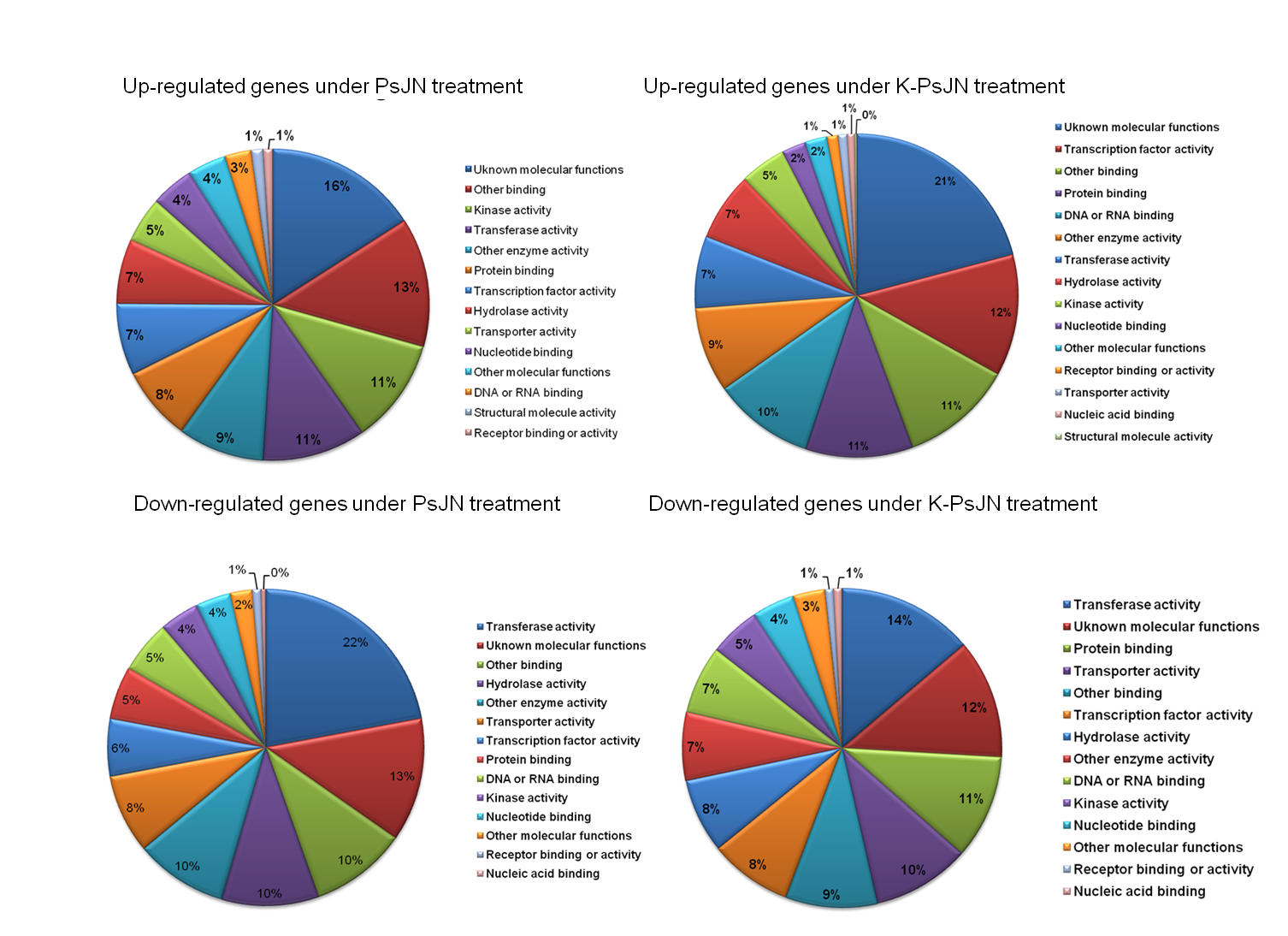

Supplement: Figure S1 — Molecular functions of the up-regulated genes or down-regulated genes under the different. (TIF) [file pone.0069435.s001.tif]

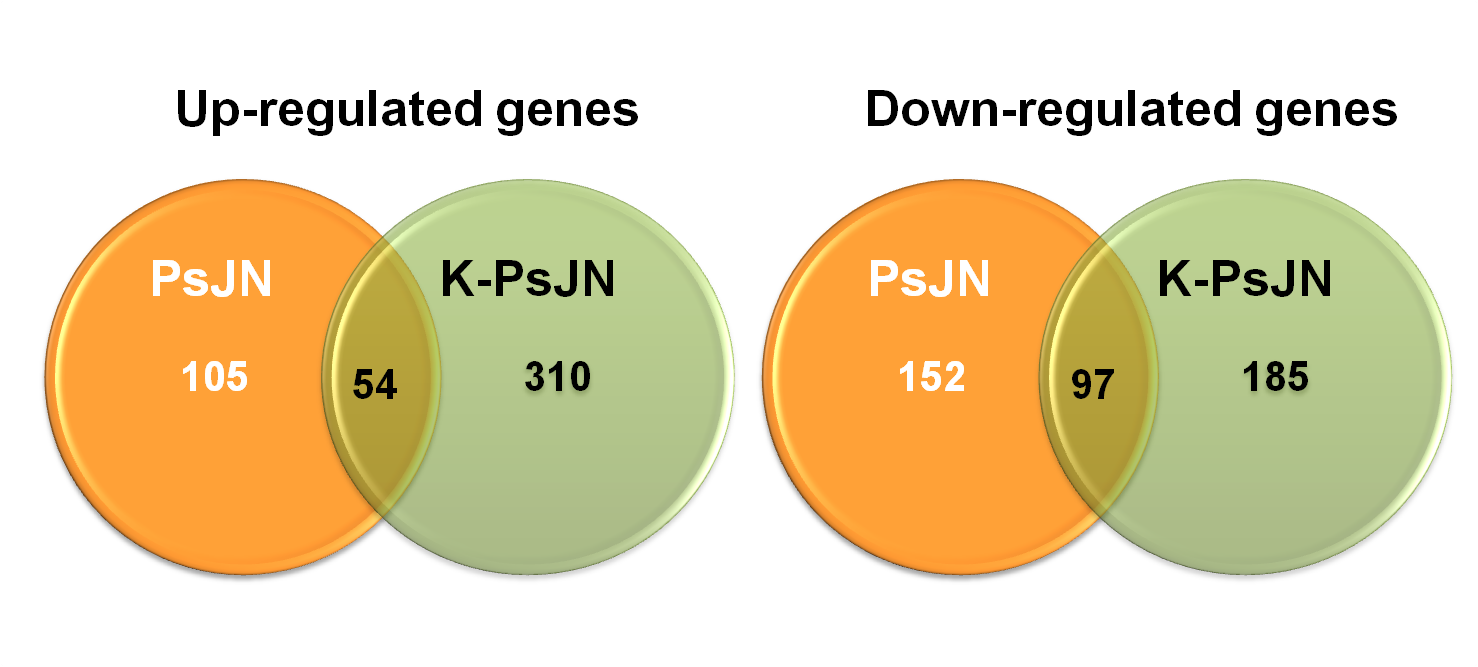

Supplement: Figure S2 — Venn diagrams of up-regulated and down-regulated genes in complete plants of 4 rosette leaves stages under PsJN or K-PsJN treatments. The intersections show the number of genes that are. (TIF) [file pone.0069435.s002.tif]

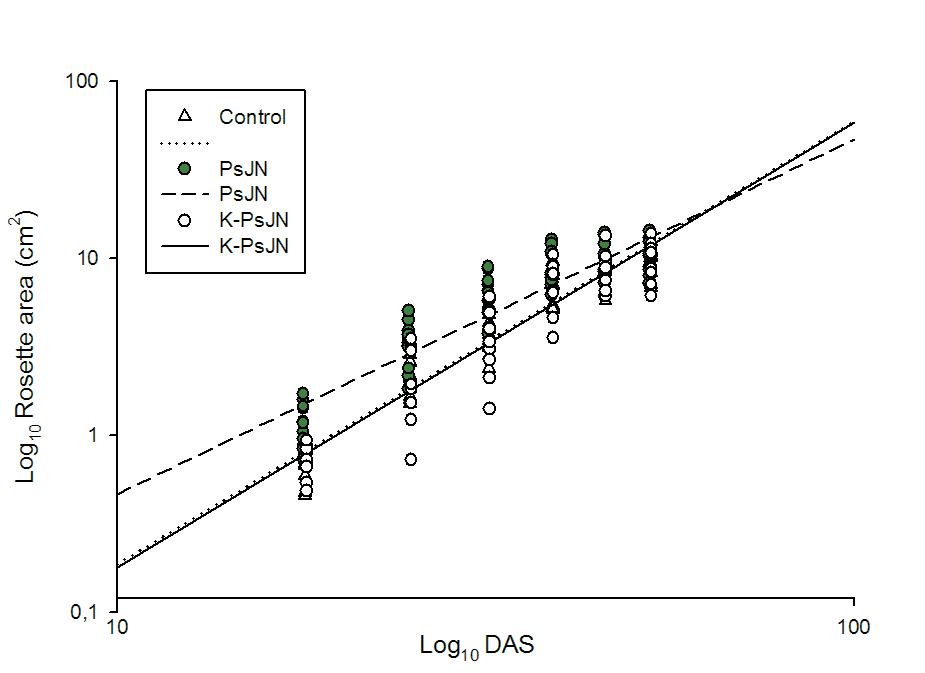

Supplement: Figure S3 — Data were Log10 transformed, and each circle or triangle represents data from one plant. (TIF) [file pone.0069435.s003.tif]
