# Supplementary material for: Effects of the Plant Growth-Promoting Bacterium Burkholderia phytofirmans PsJN throughout the Life Cycle of Arabidopsis thaliana
Source: PLoS One. 2013 Jul 15;8(7):e69435. doi: 10.1371/journal.pone.0069435 (PMC3711820; doi:10.1371/journal.pone.0069435)
Supplement: Table S1 — (DOCX) [file pone.0069435.s004.docx]

| **Table 1** Examples of Up-regulated genes by PsJN treatment belonging to different functional classifications* | | | | | | | | | | | | | | | | |
| --- | --- | --- | --- | --- | --- | --- | --- | --- | --- | --- | --- | --- | --- | --- | --- | --- |
| **ID Affymetrix** | | **Locus** | | **Name** | | **Description** | | **Fold change (Log2)** | | | ***p*-value** | | | | | |
| ***Cell cycle, organization and biogenesis*** | | | | | | | | | | | | | | | | |
| 246762_at | | AT5G27620 | | CYCH;1 | | Cyclin-dependent protein kinase | | 0.97 | | | 0.04 | | | | | |
| 252183_at | | AT3G50740 | | UGT72E1 | | UDP-glycosyltransferase/  lignin metabolic process | | 1.05 | | | 0.03 | | | | | |
| 256497_at | | AT1G31580 | | ECS1 | | Cell wall protein | | 0.92 | | | 0.04 | | | | | |
|  | |  | |  | |  | |  | | |  | | | | | |
| 245052_at | | AT2G26440 | | - | | Pectinesterase family protein | | 1.58 | | | 0.00 | | | | | |
| ***Defense response*** | |  | |  | |  | |  | | |  | | | | | |
| 261135_at | | AT1G19610 | | PDF1.4 | | PR protein | | 3.02 | | | 0.00 | | | | | |
| 266992_at | | AT2G39200 | | MLO12 | | MILDEW RESISTANCE LOCUS O 12; calmodulin binding | | 1.86 | | | 0.00 | | | | | |
| 266993_at | | AT2G39210 | | - | | Nodulin family protein | | 1.00 | | | 0.03 | | | | | |
| 245265_at | | AT4G14400 | | ACD6 | | ACD6 (ACCELERATED CELL DEATH 6); protein binding | | 1.05 | | | 0.03 | | | | | |
| **249052_at** | | **AT5G44420** | | **PDF1.2** | | **Ethylene and jasmonate responsive plant defensin** | | **1.37** | | | **0.01** | | | | | |
| 251438_s_at | | AT3G59930;AT5G33355 | | DEFL | | Defensin-like (DEFL) family proteins | | 2.87 | | | 0.00 | | | | | |
| 254805_at | | AT4G12480 | | EARLI1 | | Putative lipid transfer protein/ABA response | | 3.28 | | | 0.00 | | | | | |
| 255160_at | | AT4G07820 | | - | | Pathogenesis-related protein, putative | | 1.15 | | | 0.02 | | | | | |
| 259925_at | | AT1G75040 | | PR5 | | PATHOGENESIS-RELATED GENE 5 | | 1.71 | | | 0.00 | | | | | |
| **252618_at** | | **AT3G45140** | | **LOX2** | | **Lipoxygenase/**  **wound-induced jasmonic acid accumulation** | | **1.34** | | | **0.01** | | | | | |
| ***Development and growth*** | | | | | | | | | | | | | | | | |
| 257062_at | | AT3G18290 | | EMB2454 | | Embryo defective 2454/Embryo development/  Response to iron starvation | | 1.52 | | | 0.00 | | | |  |  |
| 267591_at | | AT2G39705 | | RTFL8 | | ROTUNDIFOLIA LIKE 8 | | 1.02 | | | 0.04 | | | |  |  |
| ***Hormone related process*** | | | | | | | | | | | | | | | | |
| 263325_at | | AT2G04240 | | XERICO | | Protein binding/Involved in ABA metabolism | | 1.52 | | | 0.00 | | | | |  |
| **245397_at** | | **AT4G14560** | | **IAA1** | | **Auxin (indole-3-acetic acid) induced gene** | | **1.07** | | | **0.02** | | | | |  |
| 250738_at | | AT5G05730 | | ASA1 | | Anthranilate synthase activity | | 1.01 | | | 0.03 | | | | |  |
| 259466_at | | AT1G19050 | | ARR7 | | Cytokinin mediated signaling pathway | | 1.52 | | | 0.00 | | | | |  |
| 259773_at | | AT1G29500 | | - | | SAUR-like auxin-responsive protein family | | 1.06 | | | 0.03 | | | | |  |
| 259783_at | | AT1G29510 | | SAUR68 | | Auxin responsive SAUR protein | | 1.01 | | | 0.04 | | | | |  |
| **261768_at** | | **AT1G15550** | | **GA3OX1** | | **Gibberellin 3-beta-dioxygenase** | | **2.38** | | | **0.00** | | | | |  |
| 263536_at | | AT2G25000 | | WRKY60* | | Transcription factor/Response to salicylic acid stimulus | | 1.14 | | | 0.02 | | | | |  |
| 251705_at | | AT3G56400 | | WRKY70* | | Activator of SA-dependent defense genes and a repressor of JA-regulated genes | | 1.11 | | | 0.03 | | | | |  |
| 259561_at | | AT1G21250 | | WAK1 | | Cell surface receptor linked signaling pathway/Response to salicylic acid stimulus | | 1.79 | | | 0.00 | | | | |  |
| ***Metabolic process*** | |  | |  | |  | |  | | |  | | | | | |
| 247524_at | | AT5G61440 | | ACHT5 | | ATYPICAL CYS HIS RICH THIOREDOXIN 5; Cell redox homeostasis | | 1.63 | | | 0.01 | | | | |  |
| 248270_at | | AT5G53450 | | ORG1 | | OBP3-responsive gene 1/ protein kinase | | 1.37 | | | 0.01 | | | | |  |
| 255025_at | | AT4G09900 | | MES12 | | METHYL ESTERASE 12/hydrolase | | 1.17 | | | 0.01 | | | | |  |
| 255381_at | | AT4G03510 | | RMA1 | | Protein binding / ubiquitin-protein ligase/ zinc ion binding | | 1.79 | | | 0.01 | | | | |  |
| 258856_at | | AT3G02040 | | SRG3 | | Senescence-related gene 3; glycerophospho  diester phospho  diesterase/ phosphoric diester hydrolase | | 1.01 | | | 0.04 | | | | |  |
| 262793_at | | AT1G13110 | | CYP71B7 | | Electron carrier/ heme binding / iron ion binding / monooxygenase/ oxygen binding | | 1.12 | | | 0.02 | | | | |  |
| 264788_at | | AT2G17880 | | - | | DNAJ heat shock protein, putative | | 1.77 | | | 0.01 | | | | |  |
| 265208_at | | AT2G36690 | | - | | Oxidoreductase, 2OG-Fe(II) oxygenase family protein | | 2.10 | | | 0.00 | | | | |  |
| ***Response to biotic or abiotic stimulus*** | | | | | |  | |  | | |  | | | | | |
| 252170_at | | AT3G50480 | | HR4 | | HOMOLOG OF RPW8 4 | | 1.52 | | | 0.00 | | | | |  |
| 252831_at | | AT4G39980 | | DHS1 | | 3-DEOXY-D-ARABINO-HEPTULOSONATE 7-PHOSPHATE SYNTHASE 1/Aromatic amino acid biosynthesis | | 1.00 | | | 0.04 | | | | |  |
| 256766_at | | AT3G22231 | | PCC1 | | PATHOGEN AND CIRCADIAN CONTROLLED 1 | | 3.05 | | | 0.00 | | | | |  |
| 267614_at | | AT2G26710 | | BAS1 | | Cytochrome p450 family and brassinosteroid homeostasis | | 1.03 | | | 0.04 | | | | |  |
| 246986_at | | AT5G67280 | | RLK | | Receptor-like kinase | | 0.98 | | | 0.03 | | | | |  |
| ***Stress response*** | |  | |  | |  | |  | | |  | | | | | |
| 246071_at | | AT5G20150 | | SPX1 | | SPX DOMAIN GENE 1/Related to Phosphate starvation | | 1.40 | | | 0.00 | | |  |  |  |
| 247540_at | | AT5G61590 | | - | | ERF/AP2 transcription factor family protein | | 1.22 | | | 0.01 | | |  |  |  |
| 256969_at | | AT3G21080 | | - | | ABC transporter-related/Response to iron starvation | | 1.10 | | | 0.02 | | |  |  |  |
| ***Transcription factors*** | |  | |  | |  | |  | | |  | | | | | |
| **245901_at** | | **AT5G11060** | | **KNAT4** | | **KNAT4 (KNOTTED1-LIKE HOMEOBOX GENE 4)/ transcription factor** | | **1.06** | | | **0.03** | |  |  |  |  |
| 250524_at | | AT5G08520 | | - | | Myb family transcription factor | | 1.00 | | | 0.04 | |  |  |  |  |
| 260070_at | | AT1G73830 | | BEE3 | | BR ENHANCED EXPRESSION 3/ transcription factor | | 1.39 | | | 0.01 | |  |  |  |  |
| 263584_at | | AT2G17040 | | ANAC036 | | Arabidopsis NAC domain containing protein 36;  Transcription factor | | 0.99 | | | 0.04 | |  |  |  |  |
| 264616_at | | AT2G17740 | | - | | DC1 domain-containing protein | | 3.33 | | | 0.00 | |  |  |  |  |
| ***Transport*** | |  | |  | |  | |  | | |  | | | | | |
| 246238_at | | AT4G36670 | | - | | Mannitol transporter, putative | | 1.79 | | | 0.00 | |  |  |  |  |
| 248062_at | | AT5G55450 | | - | | LTP family protein/Lipid transport and response to other organism | | 1.48 | | | 0.01 | |  |  |  |  |
| 248551_at | | AT5G50200 | | WR3 | | WOUND-RESPONSIVE 3; nitrate transmembrane transporter | | 1.17 | | | 0.01 | |  |  |  |  |
| 249346_at | | AT5G40780 | | LHT1 | | Amino acid transmembrane transporter | | 1.61 | | | 0.00 | |  |  |  |  |
| 250248_at | | AT5G13740 | | ZIF1 | | ZINC INDUCED FACILITATOR 1/ carbohydrate transmembrane transporter | | 1.71 | | | 0.00 | |  |  |  |  |
| 254314_at | | AT4G22470 | | - | | Protease inhibitor/seed storage/lipid transfer protein (LTP) family protein | | 2.53 | | | 0.00 | |  |  |  |  |
| ***Unknown function*** | |  | |  | |  | |  | | |  | | | | | |
| 245329_at | | AT4G14365 | | XBAT34 | | Zinc finger family protein / ankyrin repeat family protein | | 2.20 | | | 0.00 | |  |  |  |  |
| 262170_at | | AT1G74940 | | - | | Senescence-associated protein-related | | 2.02 | | | 0.00 | |  |  |  |  |
| 262331_at | | AT1G64050 | | - | | Unknown protein | | 1.22 | | | 0.01 | |  |  |  |  |
| 267384_at | | AT2G44370 | | DC1 | | DC1 domain-containing protein | | 1.38 | | | 0.00 | |  |  |  |  |
|  | |  | |  | |  | |  |  | | |  |  |  |  |  |
|  | |  | |  | |  | |  |  | | |  |  |  |  |  |
|  |  | |  | |  | |  | | |  | | | | |  |  |

* Expression changes are presented as Log_2._ Genes in bold were used for quantitative RT-PCR confirmation. Although some genes could be assigned to more than one functional classification, each gene was assigned to only one category.
