# Supplementary material for: Effects of the Plant Growth-Promoting Bacterium Burkholderia phytofirmans PsJN throughout the Life Cycle of Arabidopsis thaliana
Source: PLoS One. 2013 Jul 15;8(7):e69435. doi: 10.1371/journal.pone.0069435 (PMC3711820; doi:10.1371/journal.pone.0069435)
Supplement: Table S2 — (DOCX) [file pone.0069435.s005.docx]

| **Table 2** Examples of Down-regulated genes by PsJN treatment belonging to different functional classifications* | | | | | | | | | | |  |
| --- | --- | --- | --- | --- | --- | --- | --- | --- | --- | --- | --- |
| **ID Affymetrix** | **Locus** | | **Name** | | **Description** | | **Fold change (Log2)** | | **p-value** | |  |
| ***Cell cycle, organization and biogenesis*** | | | | | | | | | | |  |
| 260592_at | AT1G55850 | | ATCSLE1 | | Cellulose synthase/ transferase, transferring glycosyl groups | | -1.85 | | 0.00 | |  |
| 252997_at | AT4G38400 | | ATEXLA2 | | ARABIDOPSIS THALIANA EXPANSIN-LIKE A2 | | -0.93 | | 0.04 | |  |
| ***Circadian cycle*** | | | | | | | | | | |  |
| 253614_at | AT4G30350 | | - | | Heat shock protein related | | -1.13 | | 0.02 | |  |
| ***Defense*** |  | |  | |  | |  | |  | |  |
| 250670_at | AT5G06860 | | PGIP1 | | POLYGALACTURONASE INHIBITING PROTEIN 1; protein binding | | -1.34 | | 0.01 | |  |
| 252648_at | AT3G44630 | | - | | Disease resistance protein RPP1-WsB-like (TIR-NBS-LRR class), putative | | -1.06 | | 0.02 | |  |
| 252679_at | AT3G44260 | | - | | CCR4-NOT transcription complex protein, putative | | -2.14 | | 0.00 | |  |
| 259629_at | AT1G56510 | | WRR4 | | WRR4 (WHITE RUST RESISTANCE 4); ATP binding / nucleoside-triphosphatase/ nucleotide binding / transmembrane receptor | | -1.58 | | 0.00 | |  |
| 256589_at | AT3G28740 | | CYP81D1 | | CYP81D1; electron carrier/ heme binding / iron ion binding / monooxygenase/ oxygen binding | | -3.96 | | 0.00 | |  |
| ***Development and growth*** | | | | | | | | | | | |
| 247704_at | AT5G59510 | | RTFL5 | | ROTUNDIFOLIA LIKE 5 | | -1.59 | | 0.00 | |  |
| 247980_at | AT5G56860 | | GNC | | GATA, nitrate-inducible, carbon metabolism-involved); transcription factor | | -1.96 | | 0.00 | |  |
| 248622_at | AT5G49360 | | BXL1 | | BETA-XYLOSIDASE 1; hydrolase, hydrolyzing O-glycosyl compounds/seed development | | -1.43 | | 0.01 | |  |
| 255698_at | AT4G00150 | | SCL6 | | scarecrow-like transcription factor 6 (SCL6)/Cell division and differentiation | | -1.14 | | 0.02 | |  |
| 260135_at | AT1G66400 | | - | | calmodulin-related protein, putative/regulation of flower development | | -1.12 | | 0.02 | |  |
| **265742_at** | **AT2G01290** | | **RPI2** | | **ribose-5-phosphate isomerase/vegetative to reproductive phase transition of meristem** | | **-1.41** | | **0.01** | |  |
| ***Hormone related process*** | | | | | | | | | | | |
| 267357_at | AT2G40000 | | HSPRO2 | | ARABIDOPSIS ORTHOLOG OF SUGAR BEET HS1 PRO-1 2/SA response/stress | | -1.40 | | 0.01 | |  |
| 263231_at | AT1G05680 | | - | | UDP-glucoronosyl/UDP-glucosyl transferase family protein/ABA response/stress | | -2.31 | | 0.00 | |  |
| 245277_at | AT4G15550 | | IAGLU | | INDOLE-3-ACETATE BETA-D-GLUCOSYLTRANSFERASE; UDP-glycosyltransferase | | -1.97 | | 0.00 | |  |
| 245349_at | AT4G16690 | | MES16 | | METHYL ESTERASE 16; hydrolase, acting on ester bonds/methyl jasmonate esterase | | -1.22 | | 0.01 | |  |
| 248048_at | AT5G56080 | | NAS2 | | NAS2 (NICOTIANAMINE SYNTHASE 2); nicotianamine synthase/ethylene response | | -1.06 | | 0.03 | |  |
| 250420_at | AT5G11260 | | HY5 | | ELONGATED HYPOCOTYL 5/GA response/  transcription factor | | -1.64 | | 0.00 | |  |
| 261564_at | AT1G01720 | | ATAF1 | | Transcription activator/ABA pathway/defense | | -1.63 | | 0.00 | |  |
| 263184_at | AT1G05560 | | UGT75B1 | | UDP-GLUCOSYLTRANSFERASE 75B1/abscisic acid glucosyltransferase/SA response | | -2.38 | | 0.00 | |  |
| 249583_at | AT5G37770 | | TCH2 | | TCH2 (TOUCH 2); calcium ion binding/auxin and ABA response/regulation of flower development | | -1.45 | | 0.01 | |  |
| 250099_at | AT5G17300 | | - | | myb family transcription factor/involved in auxin pathway | | -1.43 | | 0.01 | |  |
| 254016_at | AT4G26150 | | CGA1 | | CYTOKININ-RESPONSIVE GATA FACTOR 1); transcription factor/negative regulation of flower development/CK and GA related | | -3.18 | | 0.00 | |  |
| 255795_at | AT2G33380 | | RD20 | | RD20 (RESPONSIVE TO DESSICATION 20); calcium ion binding/stress response/ABA and SA response | | -1.21 | | 0.02 | |  |
| 256069_at | AT1G13740 | | AFP2 | | AFP2 (ABI FIVE BINDING PROTEIN 2)/ABA response/stress response | | -0.98 | | 0.04 | |  |
| 257823_at | AT3G25190 | | - | | nodulin, putative/ethylene response | | -1.20 | | 0.02 | |  |
| 258350_at | AT3G17510 | | CIPK1 | | CIPK1 (CBL-INTERACTING PROTEIN KINASE 1); kinase/ protein binding/ABA response/stress response | | -1.25 | | 0.01 | |  |
| 258724_at | AT3G09600 | | - | | myb family transcription factor/Auxin, GA and ABA response/stress response | | -1.20 | | 0.03 | |  |
| **259980_at** | **AT1G76520** | | **-** | | **auxin efflux carrier family protein** | | **-1.09** | | **0.02** | |  |
| 266555_at | AT2G46270 | | GBF3 | | G-BOX BINDING FACTOR 3/transcription factor/ABA response | | -1.26 | | 0.01 | |  |
| 267069_at | AT2G41010 | | ATCAMBP25 | | ATCAMBP25 (ARABIDOPSIS THALIANA CALMODULIN (CAM)-BINDING PROTEIN OF 25 KDA); calmodulin binding/SA pathway | | -1.40 | | 0.01 | |  |
| 255064_at | AT4G08950 | | EXO | | EXO (EXORDIUM)/Response to brassinosteroid stimulus | | -1.32 | | 0.01 | |  |
| 258742_at | AT3G05800 | | - | | transcription factor/BR related | | -0.93 | | 0.04 | |  |
|  |  | |  | |  | |  | |  | |  |
| 261448_at | AT1G21140 | | - | | nodulin, putative/ethylene response | | -1.18 | | 0.02 | |  |
| 266296_at | AT2G29420 | | ATGSTU7 | | ATGSTU7 (ARABIDOPSIS THALIANA GLUTATHIONE S-TRANSFERASE TAU 7); glutathione transferase/  Response to SA | | -1.98 | | 0.00 | |  |
| ***Metabolic process*** | | | | | | | | | | |  |
| 245193_at | AT1G67810 | | SUFE2 | | SULFUR E 2; enzyme activator | | -1.03 | | 0.04 | |  |
| 245777_at | AT1G73540 | | ATNUDT21 | | Arabidopsis thaliana Nudix hydrolase homolog 21; hydrolase | | -1.22 | | 0.02 | |  |
| 247820_at | AT5G58380 | | SIP1 | | SIP1 (SOS3-INTERACTING PROTEIN 1); ATP binding / kinase/ protein kinase/ protein serine/threonine kinase | | -1.19 | | 0.01 | |  |
| 248207_at | AT5G53970 | | - | | aminotransferase, putative | | -1.27 | | 0.01 | |  |
| 249942_at | AT5G22300 | | NIT4 | | NITRILASE 4; 3-cyanoalanine hydratase/detoxification of nitrogen compounds | | -1.32 | | 0.01 | |  |
| 258114_at | AT3G14660 | | CYP72A13 | | CYP72A13; electron carrier/ heme binding / iron ion binding / monooxygenase/ oxygen binding | | -1.38 | | 0.01 | |  |
| 259040_at | AT3G09270 | | ATGSTU8 | | ATGSTU8 (GLUTATHIONE S-TRANSFERASE TAU 8); glutathione transferase/toxin catabolic process | | -1.14 | | 0.02 | |  |
| 259922_at | AT1G72770 | | HAB1 | | HAB1 (HOMOLOGY TO ABI1); catalytic/ protein serine/threonine phosphatase | | -1.10 | | 0.02 | |  |
| 260475_at | AT1G11080 | | SCPL31 | | scpl31 (serine carboxypeptidase-like 31); serine-type carboxypeptidase | | -0.99 | | 0.03 | |  |
| 260517_at | AT1G51420 | | SPP1 | | SPP1 (SUCROSE-PHOSPHATASE 1); catalytic/ magnesium ion binding / phosphatase/ sucrose-phosphatase | | -1.00 | | 0.03 | |  |
| 262635_at | AT1G06570 | | PDS1 | | PDS1 (PHYTOENE DESATURATION 1); 4-hydroxyphenylpyruvate dioxygenase | | -1.04 | | 0.03 | |  |
| 267492_at | AT2G30620 | | - | | histone H1.2 | | -1.13 | | 0.02 | |  |
| ***Response to biotic or abiotic stimulus*** | | | | | | | | | | |  |
| 250935_at | AT5G03240 | | UBQ3 | | UBQ3 (POLYUBIQUITIN 3); protein binding | | -0.99 | | 0.04 | |  |
| 251109_at | AT5G01600 | | ATFER1 | | ATFER1; ferric iron binding / iron ion binding | | -1.11 | | 0.03 | |  |
| 253915_at | AT4G27280 | | - | | calcium-binding EF hand family protein | | -1.89 | | 0.00 | |  |
| 266368_at | AT2G41380 | | - | | embryo-abundant protein-related | | -2.17 | | 0.00 | |  |
| ***Signal transduction*** | | | | | | | | | | |  |
| 245334_at | AT4G15800 | | RALFL33 | | Ralf-like 33); signal transducer | | -1.16 | | 0.02 | |  |
| 261319_at | AT1G53090 | | SPA4 | | SPA4 (SPA1-RELATED 4); protein binding / signal transducer | | -1.24 | | 0.01 | |  |
| ***Stress response*** | | | | | | | | | | |  |
| 247655_at | AT5G59820 | | RHL41 | | RHL41 (RESPONSIVE TO HIGH LIGHT 41); nucleic acid binding / transcription factor/ zinc ion binding | | -1.70 | | 0.00 | |  |
| 245090_at | AT2G40900 | | - | | Nodulin MtN21 family protein | | -1.10 | | 0.03 | |  |
| 247091_at | AT5G66390 | | PER72 | | peroxidase 72 (PER72) (P72) (PRXR8) | | -1.01 | | 0.04 | |  |
| 247351_at | AT5G63790 | | ANAC102 | | ANAC102 (ARABIDOPSIS NAC DOMAIN CONTAINING PROTEIN 102); transcription factor | | -2.13 | | 0.00 | |  |
| **254075_at** | **AT4G25470** | | **CBF2** | | **CBF2 (C-REPEAT/DRE BINDING FACTOR 2); DNA binding / transcription activator/ transcription factor** | | **-1.36** | | **0.01** | |  |
| 255479_at | AT4G02380 | | SAG21 | | SAG21 (SENESCENCE-ASSOCIATED GENE 21) | | -1.47 | | 0.00 | |  |
| 258677_at | AT3G08730 | | PK1 | | PK1 (PROTEIN-SERINE KINASE 1); kinase/ protein binding / protein kinase/ protein serine/threonine kinase | | -1.43 | | 0.01 | |  |
| 259432_at | AT1G01520 | | - | | myb family transcription factor | | -1.89 | | 0.00 | |  |
| **261648_at** | **AT1G27730** | | **STZ** | | **STZ (salt tolerance zinc finger); nucleic acid binding / transcription factor/ transcription repressor/ zinc ion binding** | | **-2.28** | | **0.00** | |  |
| 267168_at | AT2G37770 | | - | | aldo/keto reductase family protein | | -2.38 | | 0.00 | |  |
| ***Transcription factors*** | |  | |  | |  | |  | |  | |
| **260784_at** | **AT1G06180** | | **ATMYB13** | | **MYB DOMAIN PROTEIN 13 / transcription factor/ABA, GA, SA response/stress response** | | **-1.88** | | **0.00** | |  |
| 248246_at | AT5G53200 | | TRY | | TRY (TRIPTYCHON); DNA binding / transcription factor/thricome branching | | -1.30 | | 0.02 | |  |
| 248448_at | AT5G51190 | | - | | AP2 domain-containing transcription factor, putative | | -1.25 | | 0.01 | |  |
| 249144_at | AT5G43270 | | SPL2 | | SPL2 (SQUAMOSA PROMOTER BINDING PROTEIN-LIKE 2); transcription factor/anther development | | -0.90 | | 0.05 | |  |
| 249769_at | AT5G24120 | | SIGE | | SIGE (SIGMA FACTOR E); DNA binding / DNA-directed RNA polymerase/ sigma factor/ transcription factor | | -1.23 | | 0.01 | |  |
| 249944_at | AT5G22290 | | ANAC089 | | anac089 (Arabidopsis NAC domain containing protein 89); transcription factor | | -0.96 | | 0.05 | |  |
| 256185_at | AT1G51700 | | ADOF1 | | ADOF1; DNA binding / transcription factor | | -1.25 | | 0.01 | |  |
| 257262_at | AT3G21890 | | - | | zinc finger (B-box type) family protein | | -2.68 | | 0.00 | |  |
| 257916_at | AT3G23210 | | - | | basic helix-loop-helix (bHLH) family protein | | -1.10 | | 0.02 | |  |
| 258349_at | AT3G17609 | | HYH | | HYH (HY5-HOMOLOG); DNA binding / transcription factor | | -1.30 | | 0.01 | |  |
| 259705_at | AT1G77450 | | ANAC032 | | anac032 (Arabidopsis NAC domain containing protein 32); transcription factor | | -2.89 | | 0.00 | |  |
| 264264_at | AT1G09250 | | - | | transcription factor | | -1.55 | | 0.00 | |  |
| 265573_at | AT2G28200 | | - | | nucleic acid binding / transcription factor/ zinc ion binding | | -1.62 | | 0.00 | |  |
| 267515_at | AT2G45680 | | - | | TCP family transcription factor, putative | | -1.31 | | 0.01 | |  |
| ***Transport*** |  | |  | |  | |  | |  | |  |
| 246310_at | AT3G51895 | | SULTR3;1 | | SULFATE TRANSPORTER 3;1 | | -1.17 | | 0.02 | |  |
| 254120_at | AT4G24570 | | - | | mitochondrial substrate carrier family protein | | -2.25 | | 0.00 | |  |
| 257481_at | AT1G08430 | | ALMT1 | | ALMT1 (ALUMINUM-ACTIVATED MALATE TRANSPORTER 1); malate transmembrane transporter | | -1.79 | | 0.00 | |  |
| 258033_at | AT3G21250 | | ATMRP6 | | ATMRP6; ATPase, coupled to transmembrane movement of substances | | -1.14 | | 0.02 | |  |
| 258107_at | AT3G23560 | | ALF5 | | ALF5 (ABERRANT LATERAL ROOT FORMATION 5); antiporter/ drug transporter/ transporter | | -1.26 | | 0.01 | |  |
| 261618_at | AT1G33110 | | - | | MATE efflux family protein | | -1.15 | | 0.02 | |  |
| 262935_at | AT1G79410 | | AtOCT5 | | AtOCT5 (Arabidopsis thaliana ORGANIC CATION/CARNITINE TRANSPORTER5); carbohydrate transmembrane transporter/ sugar:hydrogen symporter | | -2.17 | | 0.00 | |  |
| ***Unknown function*** | | | | | | | | | | |  |
| 248759_at | AT5G47610 | | - | | zinc finger (C3HC4-type RING finger) family protein | | -1.07 | | 0.03 | |  |
| 249191_at | AT5G42760 | | - | | Unknown function | | -2.82 | | 0.00 | |  |
| 253830_at | AT4G27652 | | - | | unknown protein | | -3.25 | | 0.00 | |  |
| 253859_at | AT4G27657 | | - | | unknown protein | | -3.36 | | 0.00 | |  |
| 254024_at | AT4G25780 | | - | | pathogenesis-related protein, putative | | -1.07 | | 0.03 | |  |
| 254577_at | AT4G19450 | | - | | nodulin-related | | -1.03 | | 0.03 | |  |
| 250098_at | AT5G17350 | | - | | unknown protein | | -2.12 | | 0.00 | |  |
| 252010_at | AT3G52740 | | - | | unknown protein | | -1.10 | | 0.03 | |  |
| 260522_x_at | AT2G41730 | | - | | unknown protein | | -1.12 | | 0.02 | |  |
| 266965_at | AT2G39510 | | - | | nodulin MtN21 family protein | | -0.93 | | 0.04 | |  |
| 261177_at | AT1G04770 | | - | | male sterility MS5 family protein | | -1.13 | | 0.03 | |  |
|  |  | |  | |  | |  | |  | |  |
|  |  | |  | |  | |  | |  | |  |

* Expression changes are presented as Log_2._ Genes in bold were used for quantitative RT-PCR confirmation. Although some genes could be assigned to more than one functional classification, each gene was assigned to only one category.
