# Supplementary material for: Effects of the Plant Growth-Promoting Bacterium Burkholderia phytofirmans PsJN throughout the Life Cycle of Arabidopsis thaliana
Source: PLoS One. 2013 Jul 15;8(7):e69435. doi: 10.1371/journal.pone.0069435 (PMC3711820; doi:10.1371/journal.pone.0069435)
Supplement: Table S5 — Melting temperature and references, if applicable, are indicated. (DOCX) [file pone.0069435.s008.docx]

| **Locus**  **(Name)** | **Primers (5-3’)** | **Tm**  **(°C)** | **Amplicon (bp)** | **Reference** |
| --- | --- | --- | --- | --- |
| AT1G06180  (AtMYB13) | F:CCGGGCTACTTCGGTGCGGA  R:TGTTGTTGCGGAGAGGTTGGCA | 61 | 298 | Designed in the present study |
| AT1G15550  (AtGA30x1) | F:CCATTCACCTCCCACACTCT  R:GCCAGTGATGGTGAAACCTT | 61 | 401 | [[1](#_ENREF_1)] |
| AT1G27730  (STZ) | F:TCGGCGACAACCACATCCGC  R:AGTGGCACCGCTTGTGTCCG | 61 | 115 | Designed in the present study |
| AT1G76520 | F:GGAAGCTCCTCTCCGGGTGCT  R:TAACGCGCAACCAAGACGCC | 61 | 156 | Designed in the present study |
| AT2G01290  (RPI2) | F:GCCTCTTCACCGCCTCAGCC  R:TCGGTCGACGGCGTGTTTGG | 61 | 174 | Designed in the present study |
| AT3G45140  (LOX2) | F:ATCAACGCTCGTGCACGCCA  R:CCGCGGGTAAGCCTTCCTGG | 64 | 142 | Designed in the present study |
| AT4G14560  (IAA1) | F:TGGAAGTCACCAATGGGCTTAACCT  R:TGCGCTTGTTGTTGCTTCTGACG | 64 | 114 | Designed in the present study |
| AT4G25470  (CBF-2) | F: AGACCATGAGCATCCGTCGTCATA  R: CGGAATCAACCTGTGCCAAGGAAA | 61 | 100 | [[2](#_ENREF_2)] |
| AT5G11060  (KNAT4) | F:CAACACGAAAACGATGGCGTTT  R:CATCTGCCACCTCTCCGCCTCC | 61 | 371 | Designed in the present study |
| AT5G44420  (PDF1.2) | F:CTTGTTCTCTTTGCTGCTTTCGAC  R:ATGCATTACTGTTTCCGCAAACC | 61 | 106 | [[3](#_ENREF_3)] |
| AT5G61850  (LEAFY) | F:AAATGCCCCACCAAGGTGACGAAC  R:ACTCGCTCCTGATTTCTTCGCGTA | 59 | 60 | [[2](#_ENREF_2)] |
| AT1G69120  (AP1) | F:CAGACCACCCATGTTGAGAAAA  R:GCACCAAATCCAGCATCCTT | 59 | 66 | [[2](#_ENREF_2)] |

Supplementary table 1:

**REFERENCES**

1. Mitchum MG, Yamaguchi S, Hanada A, Kuwahara A, Yoshioka Y, et al. (2006) Distinct and overlapping roles of two gibberellin 3-oxidases in Arabidopsis development. Plant Journal 45: 804-818.

2. Czechowski T, Bari RP, Stitt M, Scheible WR, Udvardi MK (2004) Real-time RT-PCR profiling of over 1400 Arabidopsis transcription factors: unprecedented sensitivity reveals novel root- and shoot-specific genes. Plant Journal 38: 366-379.

3. Cartieaux F, Contesto C, Gallou A, Desbrosses G, Kopka J, et al. (2008) Simultaneous interaction of Arabidopsis thaliana with Bradyrhizobium sp strain ORS278 and Pseudomonas syriugae pv. tomato DC3000 leads to complex transcriptome changes. Molecular Plant-Microbe Interactions 21: 244-259.
